# Supplementary material for: Cerebellar growth is associated with domain-specific cerebral maturation and socio-linguistic behavior
Source: Nat Commun. 2026 May 13;17:4338. doi: 10.1038/s41467-026-72940-5 (PMC13172492; doi:10.1038/s41467-026-72940-5)
Supplement: Supplementary file 1 — Supplementary Information [file 41467_2026_72940_MOESM1_ESM.pdf]

SUPPLEMENTARY INFORMATION

CEREBELLAR GROWTH IS ASSOCIATED WITH DOMAIN-SPECIFIC CEREBRAL MATURATION AND SOCIO-LINGUISTIC BEHAVIOR

Aikaterina Manoli[1,2,3], Neville Magielse[1,2,4], Felix Hoffstaedter[2,4], Nilsu Sağlam[1], Thanos Tsigaras[2,4], Augustijn A.A. de Boer[5,6], Lorenz Ahle[1], Ceyda Yalçin[1], Milin Kim[7,8], Torgeir Moberget[7,9], Thomas Wolfers[7,10,11], Casey Paquola[2], Charlotte Grosse Wiesmann[1,12], Andre F. Marquand[5,6,13], Jörn Diedrichsen[14,15,16], Sofie L. Valk[1,2,4]

[1] Max Planck Institute for Human Cognitive and Brain Sciences, Leipzig, Germany

[2] Institute of Neuroscience and Medicine (INM-7: Brain and Behaviour), Research Center Jülich, Jülich, Germany

[3] Faculty of Medicine, Leipzig University, Leipzig, Germany

[4] Institute of Systems Neuroscience, Medical Faculty and University Hospital Düsseldorf, Heinrich Heine University, Düsseldorf, Germany

[5] Donders Institute for Brain, Cognition and Behavior, Radboud University Nijmegen, Nijmegen, The Netherlands

[6] Department for Cognitive Neuroscience, Radboud University Medical Center Nijmegen, Nijmegen, The Netherlands

[7] Centre for Precision Psychiatry, Division of Mental Health and Addiction, University of Oslo and Oslo University Hospital, Oslo, Norway

[8] Department of Psychology, Faculty of Social Sciences, University of Oslo, Oslo, Norway

[9] Department of Psychology, Pedagogy and Law, School of Health Sciences, Kristiania University College, Oslo, Norway

[10] Department of Psychiatry and Psychotherapy, University of Tübingen, Tübingen, Germany

[11] German Center for Mental Health (DZPG), Jena, Germany

[12] Cognitive Neuroscience Lab, Department of Liberal Arts and Sciences, University of Technology Nuremberg, Nuremberg, Germany

[13] Department of Neuroimaging, Institute of Psychiatry, Psychology, & Neuroscience, King's College London, London, United Kingdom

[14] Western Institute of Neuroscience, Western University, Ontario, London, Canada

[15] Department of Statistical and Actuarial Sciences, Western University, London, Ontario, Canada

[16] Department of Computer Science, Western University, London, Ontario, Canada

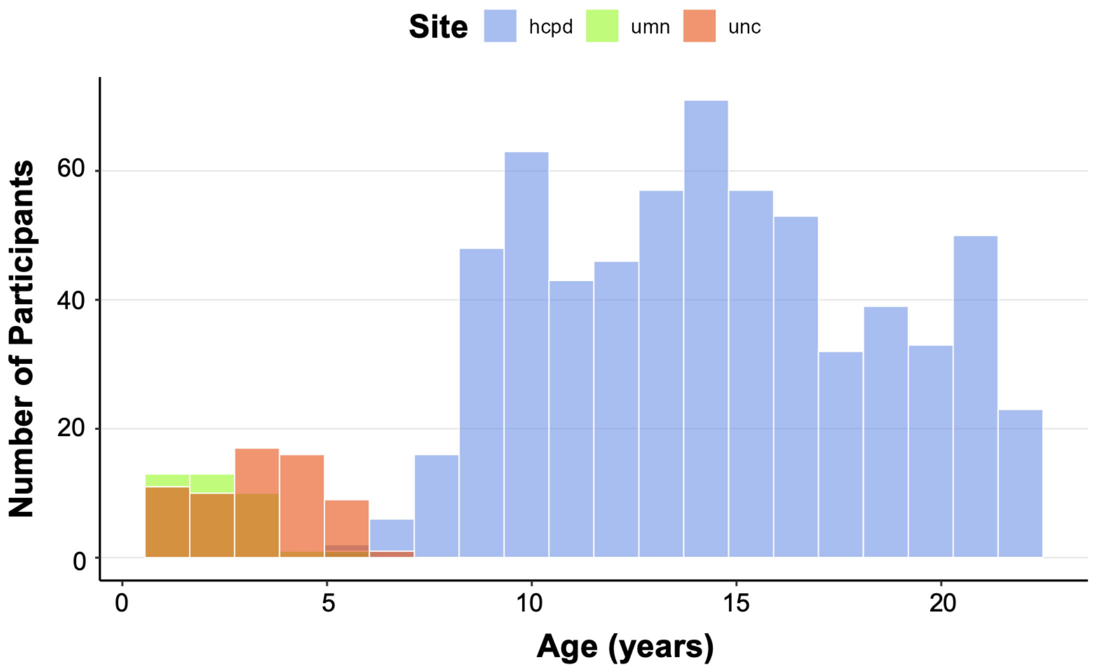

Supplementary Figure 1. Age distribution per scanner site. Source data are provided as a Source Data file. Abbreviations: hcpd = Human Connectome Project Development; umn = Baby Connectome Project University of Minnesota sample; unc = Baby Connectome Project University of North Carolina sample.

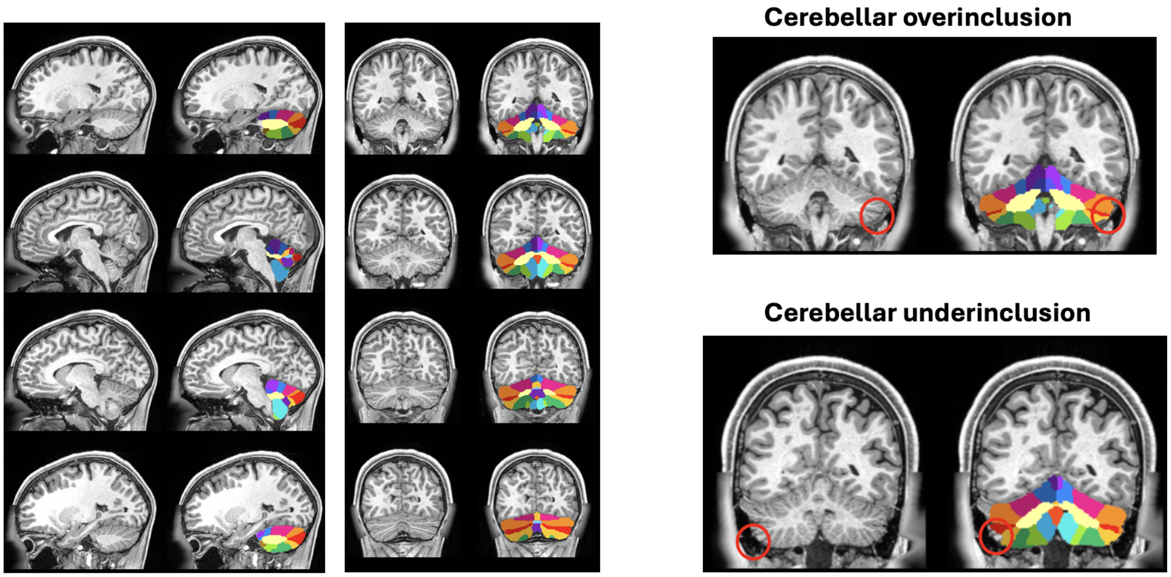

Supplementary Figure 2. Example lobular segmentations performed with the ACAPULCO algorithm. Left: Example ACAPULCO segmentation, with a native space lobular mask overlaid on a participant's T1-weighted image (sagittal and coronal views). Right: Example of cerebellar overinclusion (top; circled in red) and underinclusion (bottom; circled in red). For both types of segmentation errors, slice-wise manual correction of the mask was performed by tracing cerebellar fissures and folia (see **Methods: Cerebellar parcellation**). In the case of overinclusion, the ACAPULCO mask included parts of the cerebral cortex, non-brain infratentorial tissue, and/or lower skull and neck tissue. In the case of underinclusion, the ACAPULCO mask often missed parts of the cerebellar grey matter. Both errors were most common around postero-lateral cerebellar regions.

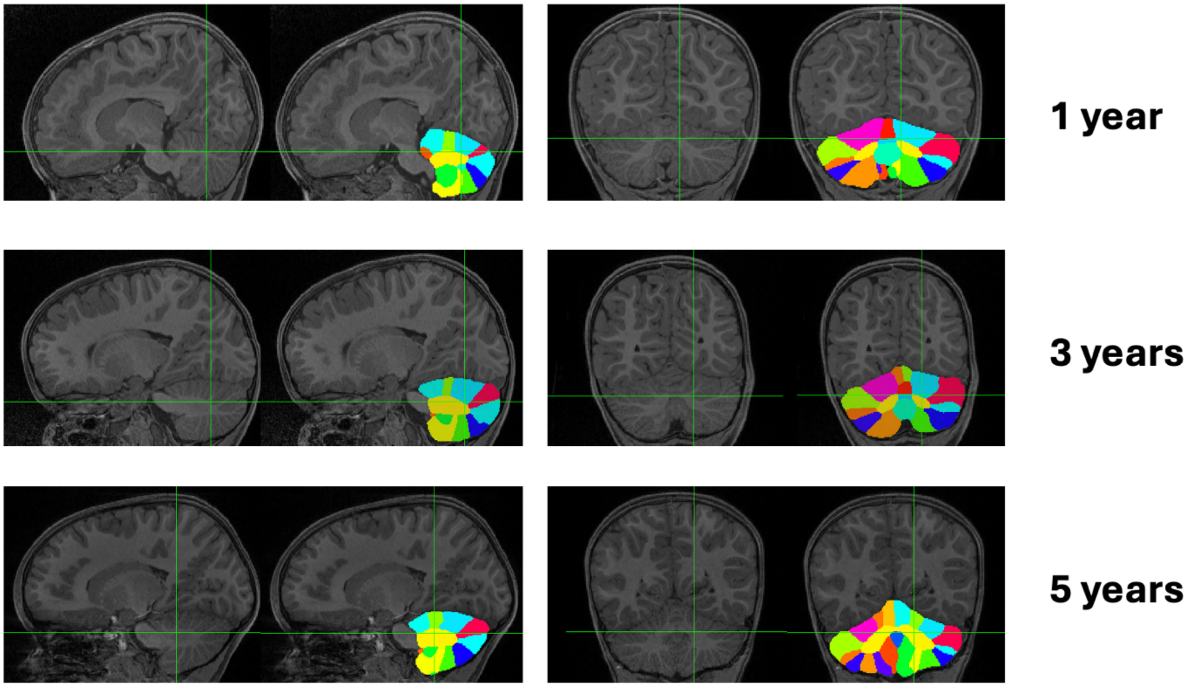

Supplementary Figure 3. Example lobular segmentations performed with the ACAPULCO algorithm in the BCP dataset (see **Methods: Cerebellar parcellation**). Native space ACAPULCO segmentation masks are overlaid on participants' T1-weighted images (sagittal and coronal views) in example timepoints (1, 3, and 5 years) across the BCP age range (1-5 years).

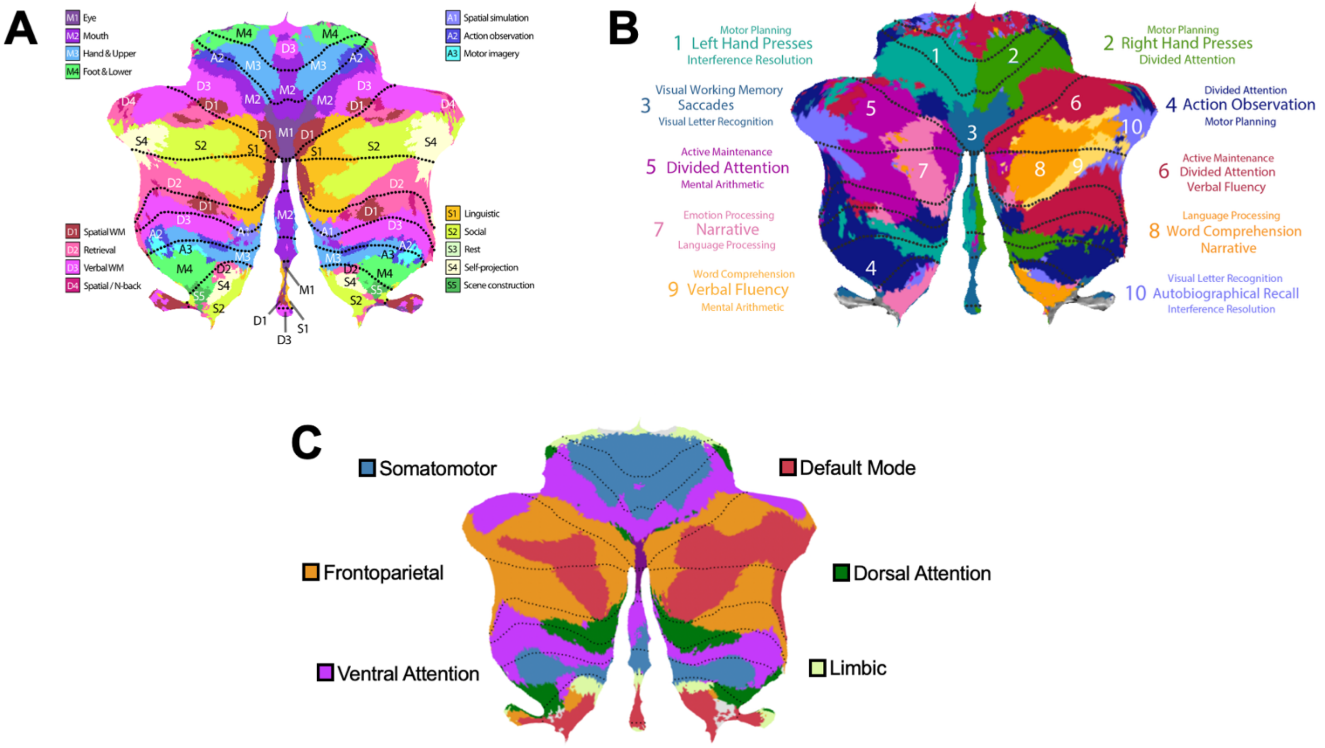

Supplementary Figure 4. Functional cerebellar parcellations. **A.** Functional fusion atlas, adapted with permission from Nettekoven et al. (2024). **B.** MDTB atlas, adapted with permission from King et al. (2019). **C.** Resting-state atlas, based on Buckner et al. (2011) and plotted via the openly available cerebellar atlas viewer (diedrichsenlab.org/imaging/AtlasViewer/index.htm).

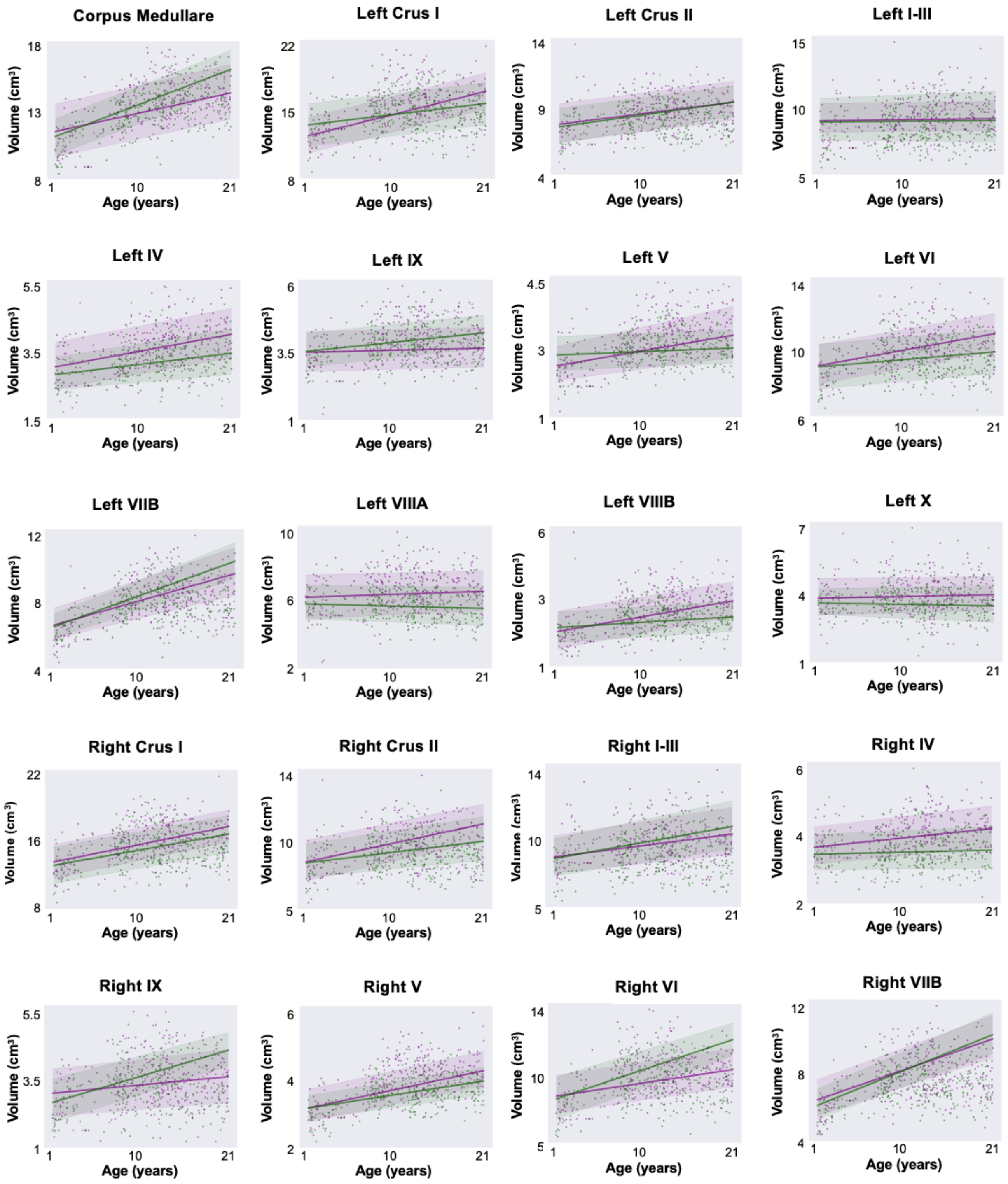
Corpus Medullare
18
13
8
Volume (cm³)
1 10 21
Age (years)
Left Crus I
22
15
8
Volume (cm³)
1 10 21
Age (years)
Left Crus II
14
9
4
Volume (cm³)
1 10 21
Age (years)
Left I-III
15
10
5
Volume (cm³)
1 10 21
Age (years)
Left IV
5.5
3.5
1.5
Volume (cm³)
1 10 21
Age (years)
Left IX
6
3.5
1
Volume (cm³)
1 10 21
Age (years)
Left V
4.5
3
1
Volume (cm³)
1 10 21
Age (years)
Left VI
14
10
6
Volume (cm³)
1 10 21
Age (years)
Left VIIB
12
8
4
Volume (cm³)
1 10 21
Age (years)
Left VIIIA
10
6
2
Volume (cm³)
1 10 21
Age (years)
Left VIIIB
6
3
1
Volume (cm³)
1 10 21
Age (years)
Left X
7
4
1
Volume (cm³)
1 10 21
Age (years)
Right Crus I
22
16
8
Volume (cm³)
1 10 21
Age (years)
Right Crus II
14
10
5
Volume (cm³)
1 10 21
Age (years)
Right I-III
14
10
5
Volume (cm³)
1 10 21
Age (years)
Right IV
6
4
2
Volume (cm³)
1 10 21
Age (years)
Right IX
5.5
3.5
1
Volume (cm³)
1 10 21
Age (years)
Right V
6
4
2
Volume (cm³)
1 10 21
Age (years)
Right VI
14
10
5
Volume (cm³)
1 10 21
Age (years)
Right VIIB
12
8
4
Volume (cm³)
1 10 21
Age (years)

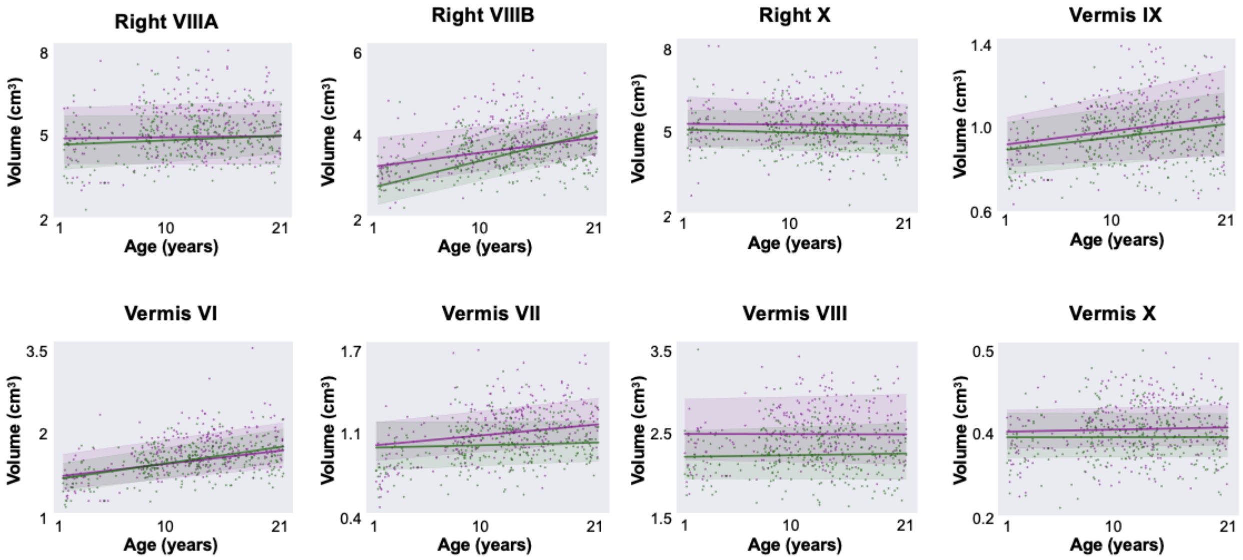

Supplementary Figure 5. Sex-stratified normative trajectories for all lobules in the ACAPULCO segmentation. Bold lines represent the mean trajectory per sex. Shaded areas represent the 68% confidence interval. Source data are provided as a Source Data file. Purple: male; green: female.

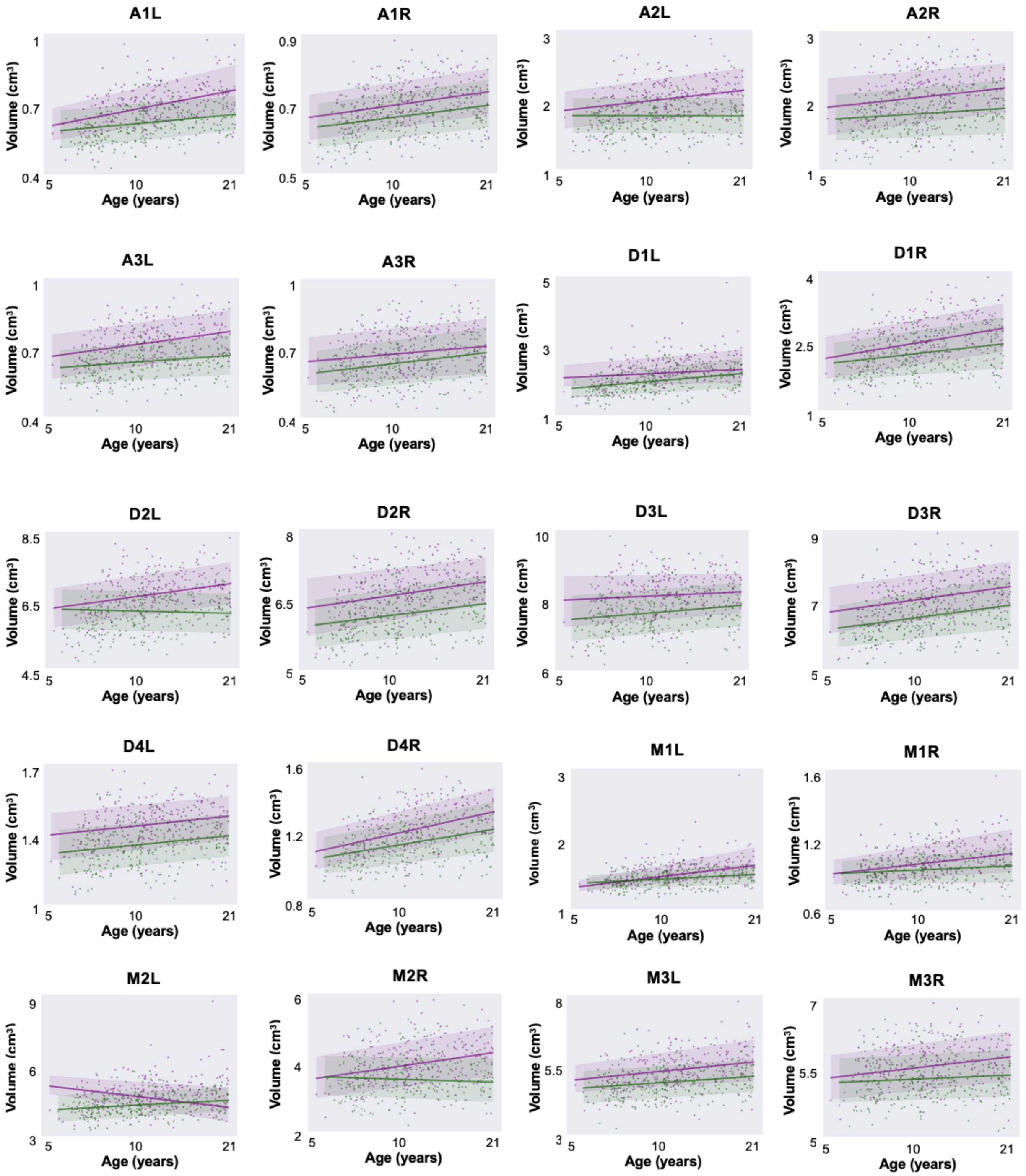
A1L
Volume (cm³)
1
0.7
0.4
5
10
21
Age (years)
A1R
Volume (cm³)
0.9
0.7
0.5
5
10
21
Age (years)
A2L
Volume (cm³)
3
2
1
5
10
21
Age (years)
A2R
Volume (cm³)
3
2
1
5
10
21
Age (years)
A3L
Volume (cm³)
1
0.7
0.4
5
10
21
Age (years)
A3R
Volume (cm³)
1
0.7
0.4
5
10
21
Age (years)
D1L
Volume (cm³)
5
3
1
5
10
21
Age (years)
D1R
Volume (cm³)
4
2.5
1
5
10
21
Age (years)
D2L
Volume (cm³)
8.5
6.5
4.5
5
10
21
Age (years)
D2R
Volume (cm³)
8
6.5
5
5
10
21
Age (years)
D3L
Volume (cm³)
10
8
6
5
10
21
Age (years)
D3R
Volume (cm³)
9
7
5
5
10
21
Age (years)
D4L
Volume (cm³)
1.7
1.4
1
5
10
21
Age (years)
D4R
Volume (cm³)
1.6
1.2
0.8
5
10
21
Age (years)
M1L
Volume (cm³)
3
2
1
5
10
21
Age (years)
M1R
Volume (cm³)
1.6
1.2
0.6
5
10
21
Age (years)
M2L
Volume (cm³)
9
6
3
5
10
21
Age (years)
M2R
Volume (cm³)
6
4
2
5
10
21
Age (years)
M3L
Volume (cm³)
8
5.5
3
5
10
21
Age (years)
M3R
Volume (cm³)
7
5.5
5
5
10
21
Age (years)

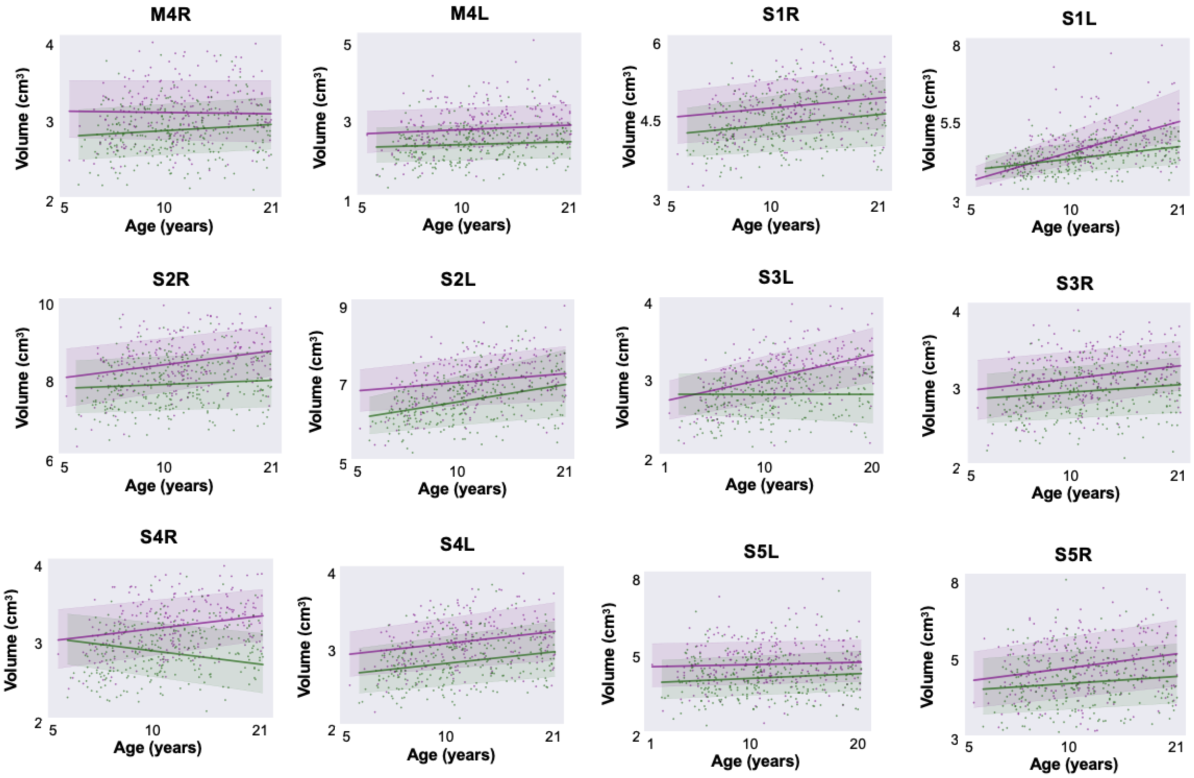

Supplementary Figure 6. Sex-stratified normative trajectories for all parcels in the functional fusion atlas. Bold lines represent the mean trajectory per sex. Shaded areas represent the 68% confidence interval. Source data are provided as a Source Data file. Purple: male; Green: female. Abbreviations: A = Action; D = Demand; M = Motor; S = Socio-Linguistic; L = left; R = right.

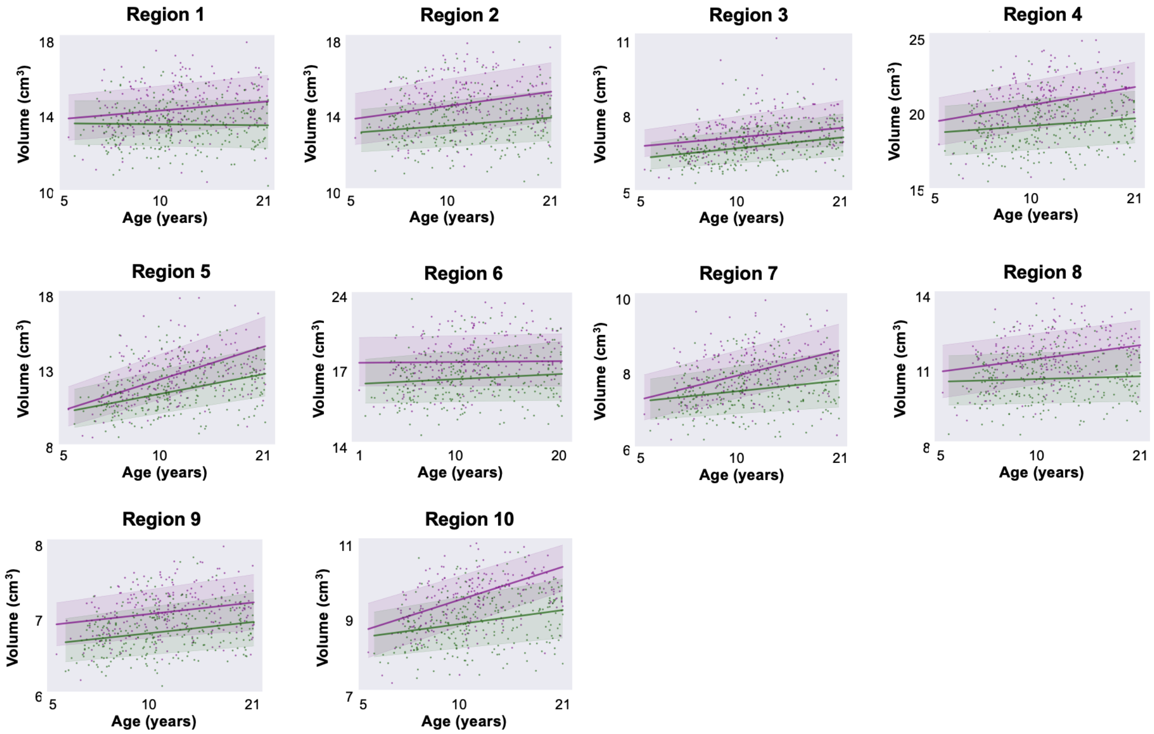

Supplementary Figure 7. Sex-stratified normative trajectories for all parcels in the MDTB atlas. Bold lines represent the mean trajectory per sex. Shaded areas represent the 68% confidence interval. Source data are provided as a Source Data file. Purple: male; green: female.

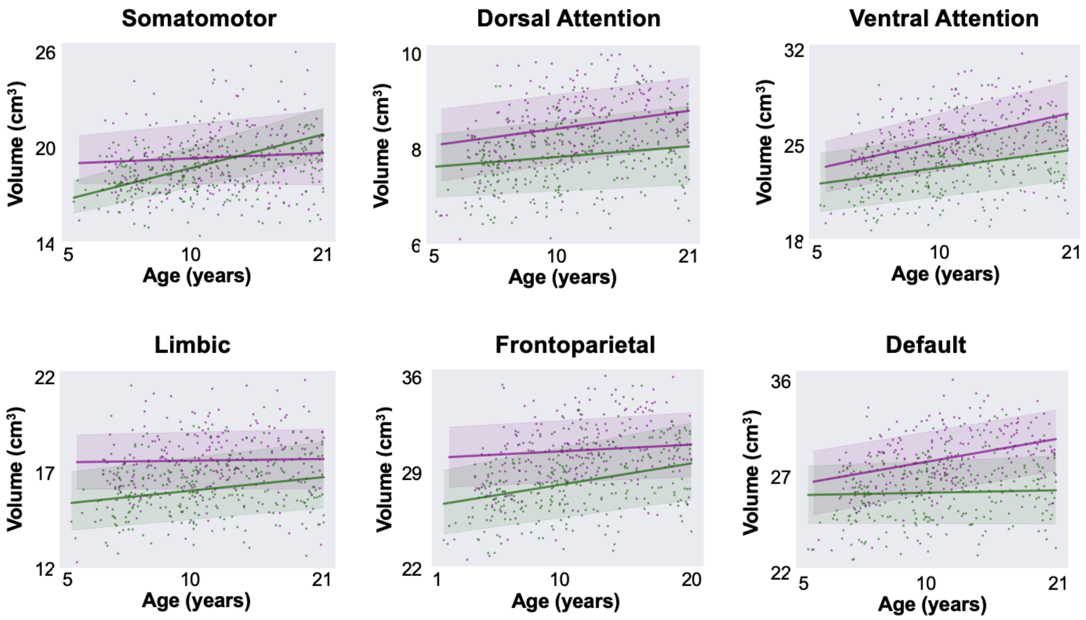

Supplementary Figure 8. Sex-stratified normative trajectories for all parcels in the resting-state atlas. Bold lines represent the mean trajectory per sex. Shaded areas represent the 68% confidence interval. Source data are provided as a Source Data file. Purple: male; green: female.

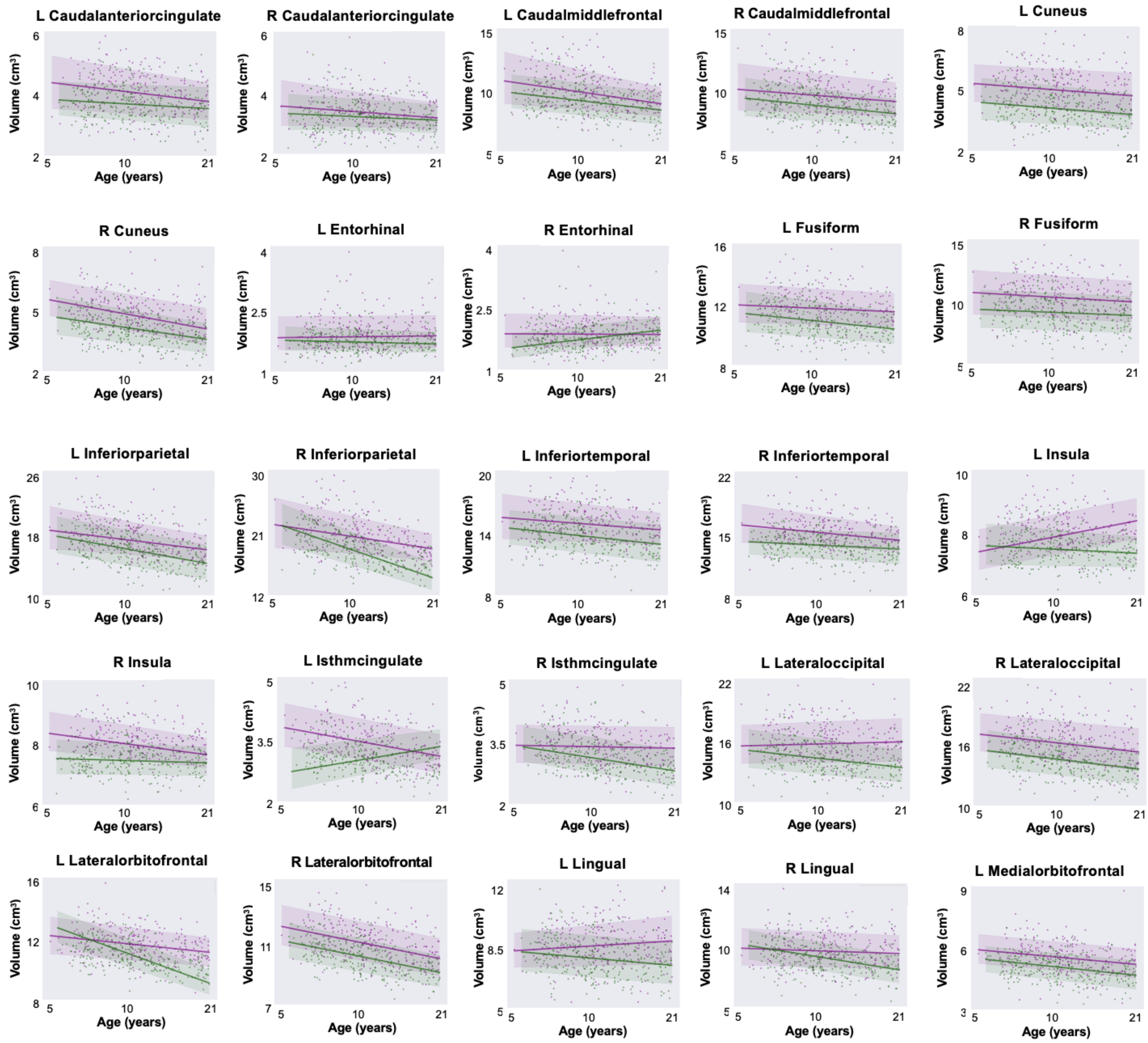
L Caudalanteriorcingulate
R Caudalanteriorcingulate
L Caudalmiddlefrontal
R Caudalmiddlefrontal
L Cuneus
R Cuneus
L Entorhinal
R Entorhinal
L Fusiform
R Fusiform
L Inferiorparietal
R Inferiorparietal
L Inferiortemporal
R Inferiortemporal
L Insula
R Insula
L Isthmcingulate
R Isthmcingulate
L Lateraloccipital
R Lateraloccipital
L Lateralorbitofrontal
R Lateralorbitofrontal
L Lingual
R Lingual
L Medialorbitofrontal
Volume (cm³)
Age (years)

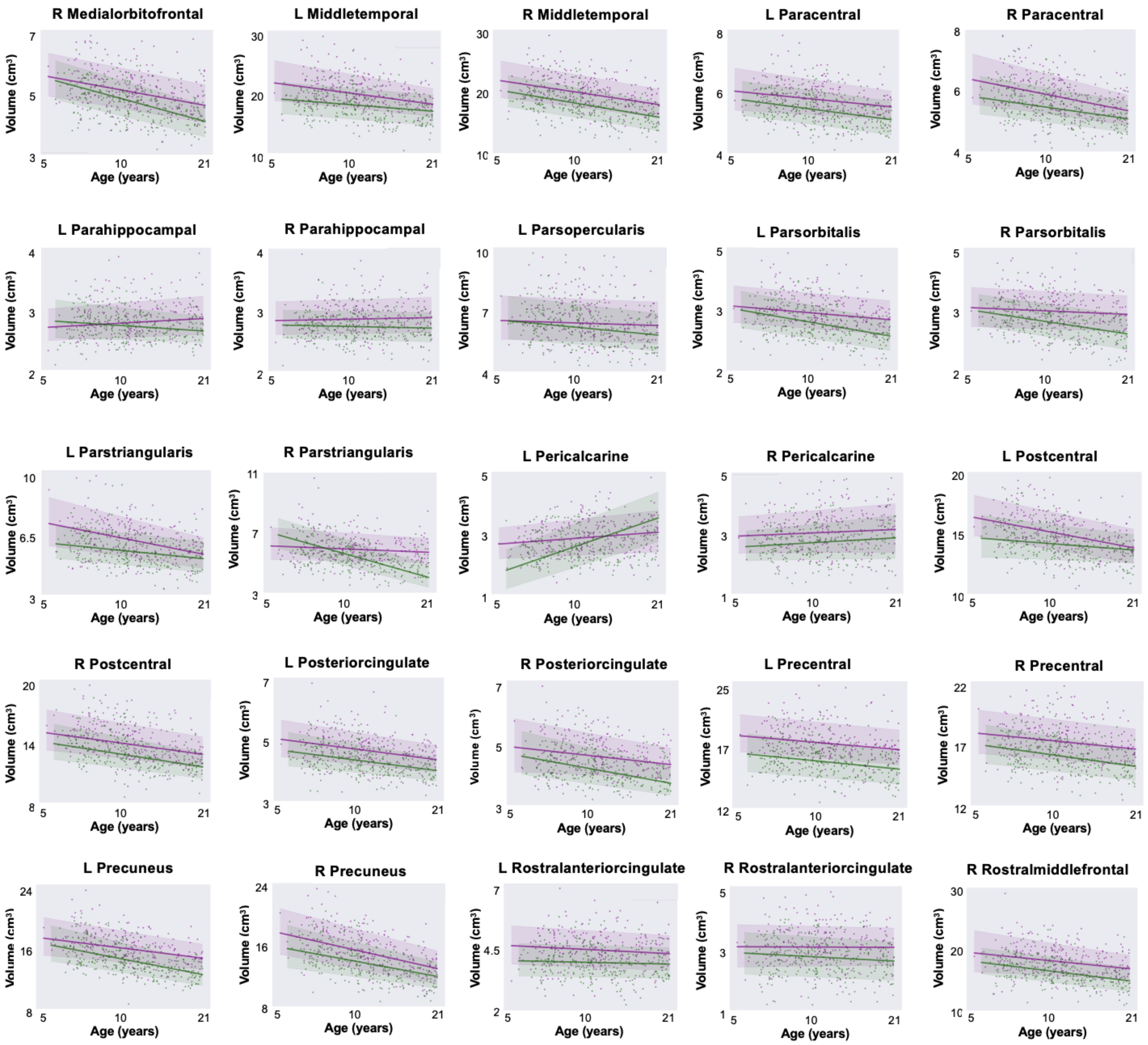
R Medialorbitofrontal
L Middletemporal
R Middletemporal
L Paracentral
R Paracentral
L Parahippocampal
R Parahippocampal
L Parsopercularis
L Parsorbitalis
R Parsorbitalis
L Parstriangularis
R Parstriangularis
L Pericalcarine
R Pericalcarine
L Postcentral
R Postcentral
L Posteriorcingulate
R Posteriorcingulate
L Precentral
R Precentral
L Precuneus
R Precuneus
L Rostralanteriorcingulate
R Rostralanteriorcingulate
R Rostralmiddlefrontal
Volume (cm³)
Age (years)

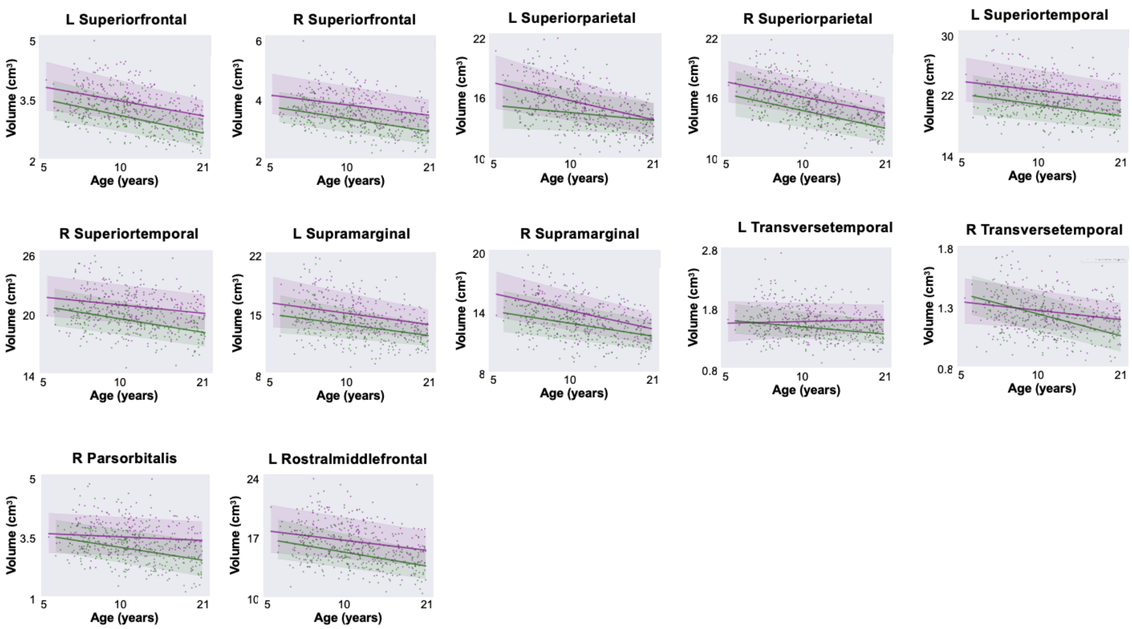

Supplementary Figure 9. Sex-stratified normative trajectories for all parcels in the DK atlas. Bold lines represent the mean trajectory per sex. Shaded areas represent the 68% confidence interval. Source data are provided as a Source Data file. Purple: male; green: female. Abbreviations: L = left; R = right.

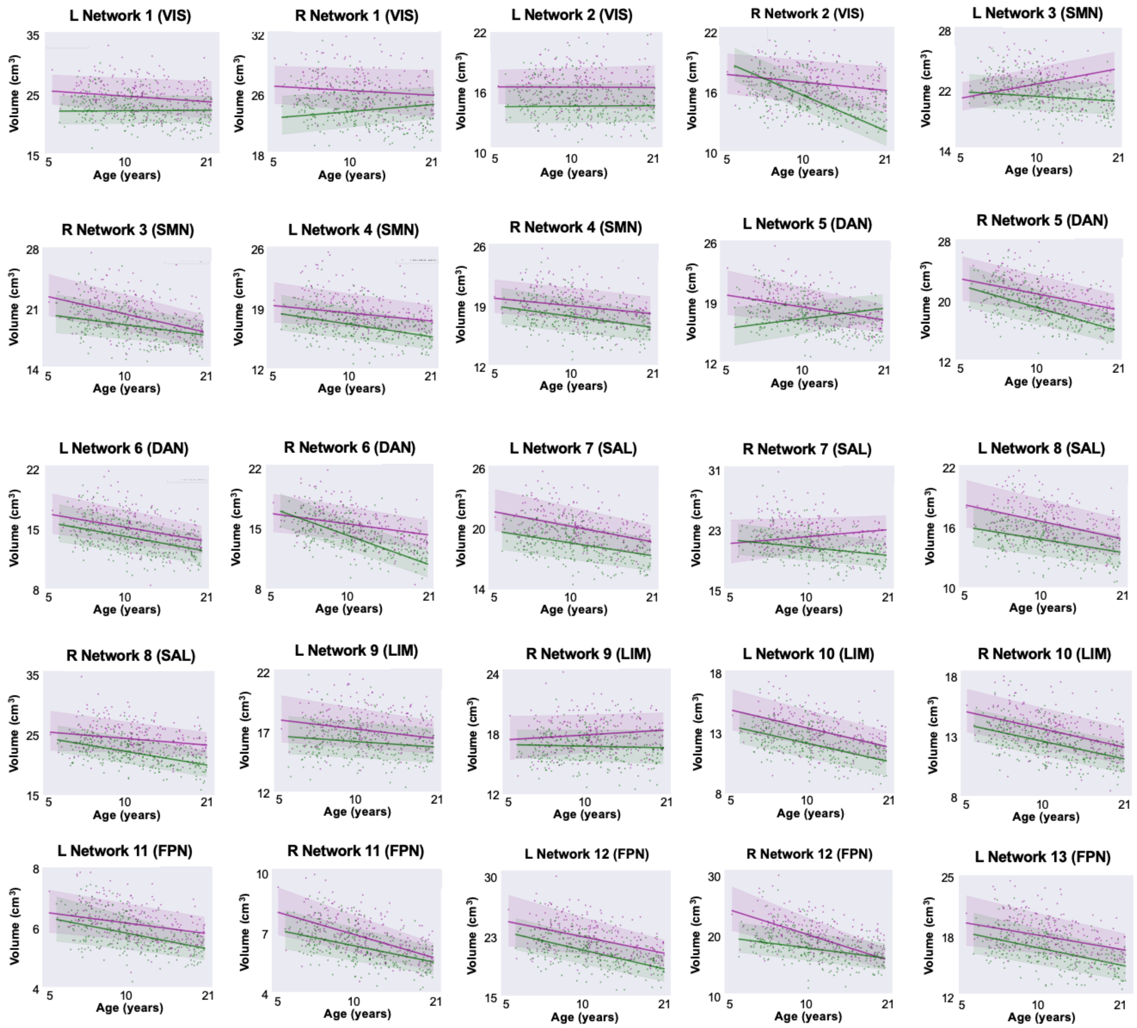
L Network 1 (VIS)
R Network 1 (VIS)
L Network 2 (VIS)
R Network 2 (VIS)
L Network 3 (SMN)
R Network 3 (SMN)
L Network 4 (SMN)
R Network 4 (SMN)
L Network 5 (DAN)
R Network 5 (DAN)
L Network 6 (DAN)
R Network 6 (DAN)
L Network 7 (SAL)
R Network 7 (SAL)
L Network 8 (SAL)
R Network 8 (SAL)
L Network 9 (LIM)
R Network 9 (LIM)
L Network 10 (LIM)
R Network 10 (LIM)
L Network 11 (FPN)
R Network 11 (FPN)
L Network 12 (FPN)
R Network 12 (FPN)
L Network 13 (FPN)
Volume (cm³)
Age (years)

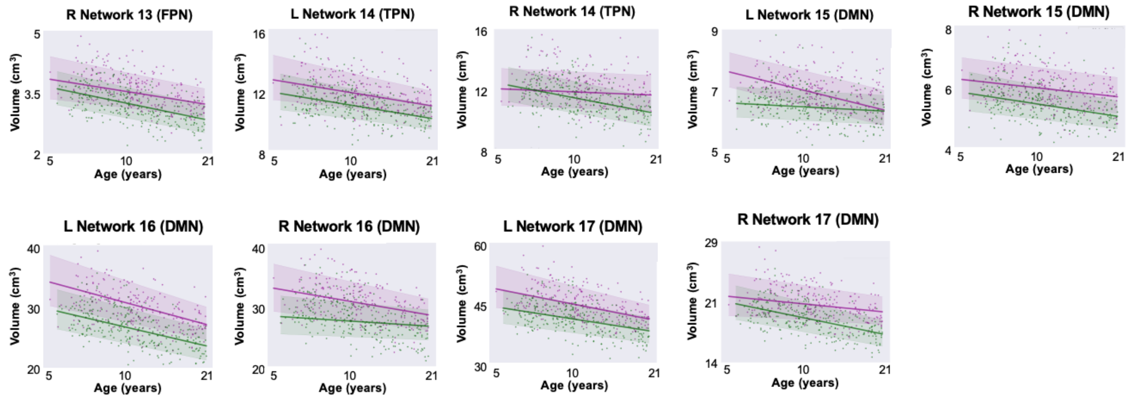

Supplementary Figure 10. Sex-stratified normative trajectories for all parcels in the Yeo et al. 17-network atlas. Bold lines represent the mean trajectory per sex. Shaded areas represent the 68% confidence interval. Source data are provided as a Source Data file. Purple: male; green: female. Abbreviations: VIS = visual network; SMN = somatomotor network; DAN = dorsal attention network; SAL = salience network; LIM = limbic network; FPN = frontoparietal network; TPN = temporoparietal network; DMN = default mode network; L = left; R = right.

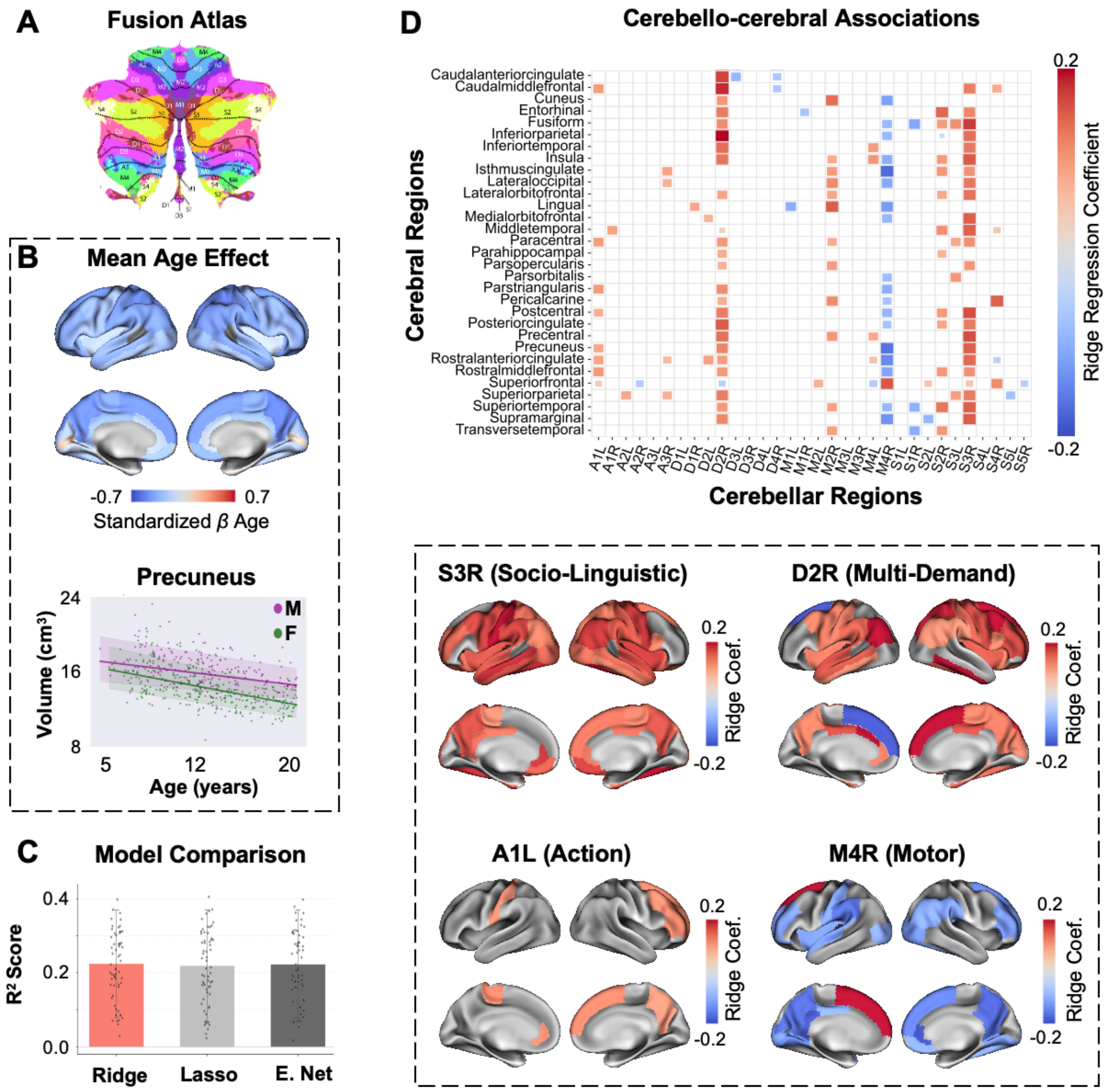

Supplementary Figure 11. Associations of cerebellar (fusion atlas) and cerebral (DK atlas) growth trajectories. **A.** Functional fusion atlas (adapted with permission from Nettekoven et al.[6]). **B.** Mean effect of age on the DK atlas (top) and normative trajectory of precuneus (bottom). Bold lines represent the mean trajectories for each sex. Shaded areas represent the 68% CI. **C.** Comparison of Ridge, Lasso, and ElasticNet regularization models based on average R^2 scores (10-fold cross-validation). The Ridge model marginally outperformed the other two and was selected (mean R^2= .22). Data points denote parcel–level cross-validation performance. Error bars represent the standard error of the mean. **D.** Top: Significant cerebello-cerebral associations (10,000 permutations, FDR corrected at q = .05). A global α = 100 was selected for associations based on a multi-output Lasso model predicting all cerebral parcels simultaneously and aligns with the distribution of parcel-wise optimal α values, where over 80% of cerebral parcels individually favored α = 100. Left and right DK parcels in the heatmap are averaged for brevity (see **Supplementary Figure 12** for left and right parcel weights). The

median Spearman correlation between homologous left–right parcel pairs was r = .71, with an interquartile range of .61–.81. Bottom: FDR-corrected weights of cerebellar parcels with the largest number of significant associations per functional domain in the fusion atlas (i.e., socio-linguistic, multi-demand, action, motor), projected on the DK atlas. Note that, unlike in the heatmap, cerebral hemispheres are not averaged in this plot. Source data are provided as a Source Data file. Abbreviations: Coef. = coefficient; E. Net = ElasticNet; M = male; F = female; L = left; R = right.

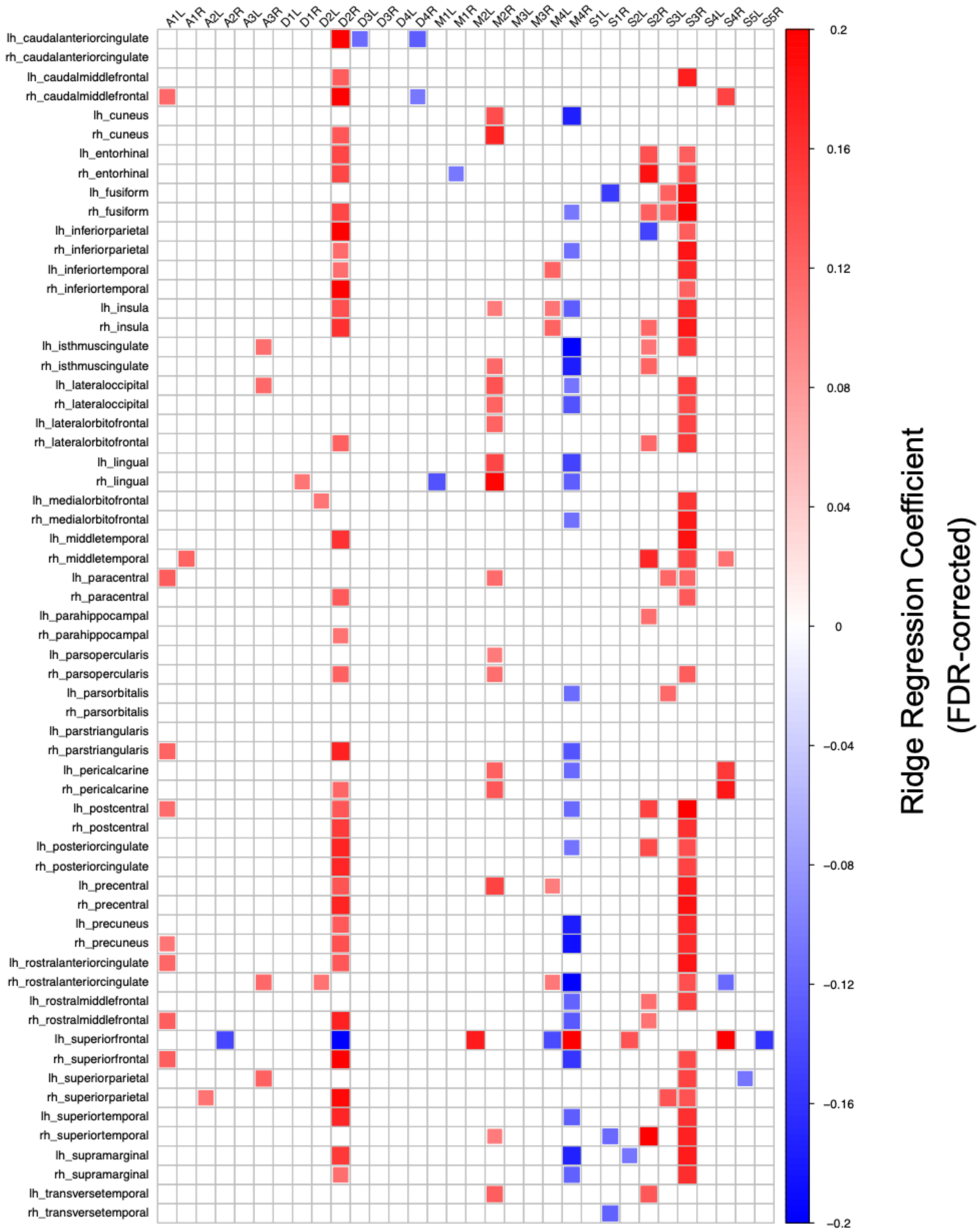

Supplementary Figure 12. Associations of cerebellar (fusion atlas) and cerebral (DK) growth trajectories per DK hemisphere. The heatmap shows significant cerebro-cerebellar associations (10,000 permutations, false discovery rate (FDR)-corrected at q = .05). Abbreviations: lh = left hemisphere; rh = right hemisphere.

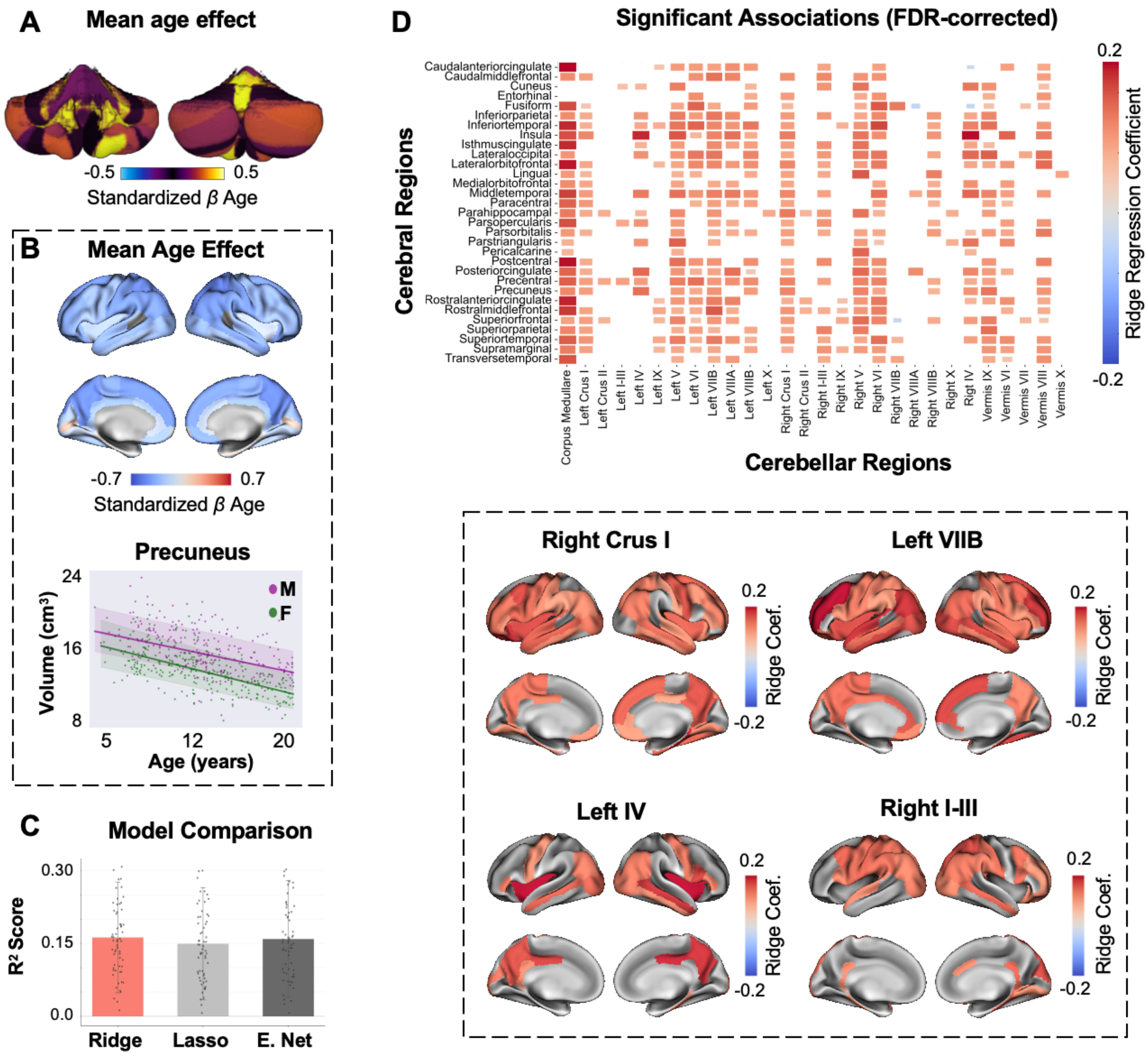

Supplementary Figure 13. Associations of cerebellar and cerebral growth trajectories. **A.** Mean effect of age on growth per anatomical lobule (HCP-D dataset; 5-21 years). **B.** Mean effect of age on growth per DK parcel (top) and an example normative trajectory for the precuneus (bottom). Bold lines represent the mean trajectory per sex. Shaded areas represent the 68% confidence interval. **C.** Comparison of Ridge, Lasso, and ElasticNet regularization models based on R^2 scores (ten-fold cross-validation). The Ridge model marginally outperformed the other two and was selected (mean R^2= .16). Data points denote parcel–level cross-validation performance. Error bars represent the standard error of the mean. **D.** Top: Significant cerebro-cerebellar associations (10,000 permutations, false discovery rate (FDR)-corrected at q = .05). A global α = 1,000 was selected for associations based on a multi-output Ridge model predicting all cerebral parcels simultaneously and aligns with the distribution of parcel-wise optimal α values, where over 80% of cerebral parcels individually favored α = 1,000. Left and right DK parcels in the heatmap are averaged for brevity. The median Spearman correlation between

homologous left–right parcel pairs was r = .70, with an interquartile range of .60–.80. Bottom: FDR-corrected weights for example anterior (lobules I-III and IV) and posterior (Right Crus I and Left VIIB) cerebellar parcels, projected on the DK atlas. Note that, unlike in the heatmap, cerebral hemispheres are not averaged in this plot. Source data are provided as a Source Data file. Abbreviations: Coef. = coefficient; M = male; F = female; E. Net = ElasticNet.

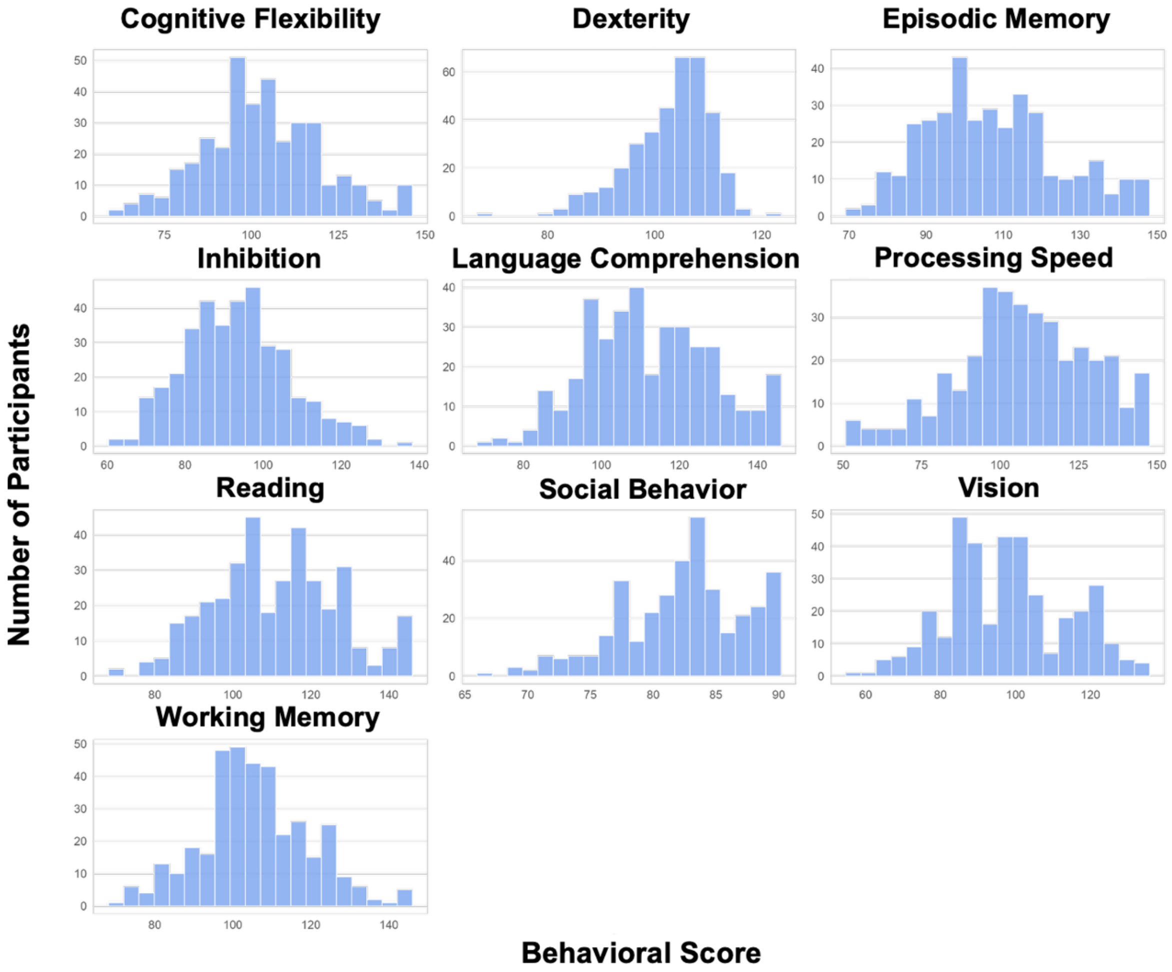

Supplementary Figure 14. Distribution of participants' scores of the selected behavioral tasks in the Human Connectome Project Development dataset (N = 457; 5-21 years; participants without behavioral scores were removed). Source data are provided as a Source Data file.

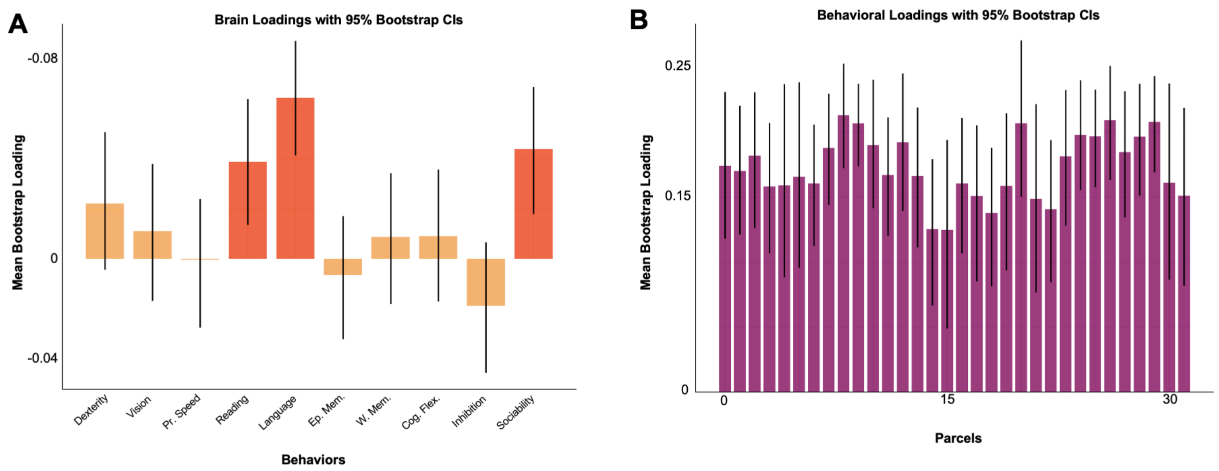

Supplementary Figure 15. Bootstrap resampling of PLS loadings. Bootstrap resampling (10,000 iterations) results for behavioral scores **(A)** and functional fusion parcels **(B)**. Bars correspond to 95% confidence intervals. Source data are provided as a Source Data file. Abbreviations: Pr. Speed = Processing Speed; Ep. Mem. = Episodic Memory; W. Mem. = Working Memory; Cog. Flex. = Cognitive Flexibility.

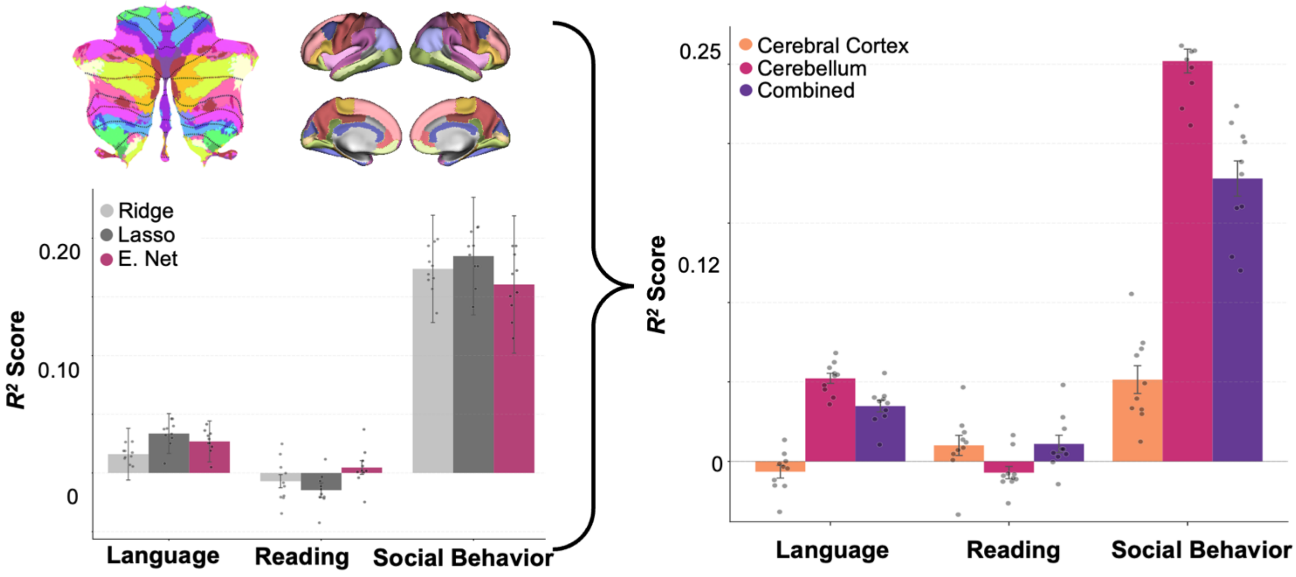

Supplementary Figure 16. Cerebellar (fusion), cerebral (DK), and combined cerebello-cerebral parcel *z*-scores were compared for how well they predicted socio-linguistic behaviors using ElasticNet regularization. Left: Mean Lasso, Ridge, and ElasticNet model performance (R^2) across cerebellar, cerebral, and combined models for each behavior, averaged over 10-fold cross-

validation. Error bars represent the standard error of the mean. Right: ElasticNet model performance (R^2) for cerebellum-only, cerebral-only, and combined model across language, reading abilities and social behavior, averaged over repeated 10-fold cross-validation. Source data are provided as a Source Data file. Data points denote repeat-level cross-validation performance. Error bars represent the standard error of the mean. Abbreviations: E. Net = ElasticNet.

Supplementary Table 1. Sex-stratified means of standardized β_{age} across lobular regions

Lobular Regions	Male (Mean [95% CI])	Female (Mean [95% CI])
Corpus Medullare	0.46 [-0.08 1.04]	0.98 [0.26 1.84]
Left Crus I	0.40 [-0.22 0.91]	0.34 [-0.21 0.99]
Left Crus II	0.36 [-0.15 0.97]	0.35 [-0.27 1.38]
Left I-III	0.03 [-0.22 0.25]	0.02 [-0.54 0.63]
Left IV	0.25 [-0.15 0.66]	0.07 [-0.30 0.44]
Left IX	0.04 [-0.64 0.67]	0.42 [-0.27 1.24]
Left V	0.41 [-0.22 0.89]	0.07[-0.13 0.87]
Left VI	0.42 [-0.23 1.09]	0.26 [-0.37 0.91]
Left VIIB	0.61 [0.01 1.17]	0.65 [0.34 1.87]
Left VIIIA	0.08 [-0.42 0.59]	-0.08 [-0.54 0.36]
Left VIIIB	0.40 [-0.02 0.85]	0.20 [-0.34 0.72]
Left X	0.05 [-0.45 0.51]	-0.05 [-0.47 0.36]
Right Crus I	0.48 [-0.10 1.02]	0.45 [-0.17 1.10]
Right Crus II	0.40 [-0.14 1.23]	0.34 [-0.20 1.11]
Right I-III	0.26 [-0.20 0.81]	0.29 [-0.09 1.89]
Right IX	0.13 [-0.49 0.78]	0.32 [-0.17 1.65]
Right V	0.49 [0.06 0.91]	0.41 [-0.08 0.93]
Right VI	0.32 [-0.27 0.86]	0.62 [-0.05 2.01]
Right VIIB	0.78 [0.17 1.48]	0.79 [0.29 1.97]
Right VIIIA	0.05 [-0.60 0.57]	0.05 [-0.40 0.64]
Right VIIIB	0.29 [-0.19 0.76]	0.32 [-0.24 0.87]
Right X	-0.02 [-0.52 0.48]	-0.08 [-0.43 0.29]
Right IV	0.40 [-0.15 0.80]	0.32 [-0.12 0.82]
Vermis IX	0.28 [-0.17 0.72]	0.25 [-0.27 0.87]
Vermis VI	0.34 [-0.15 0.78]	0.34 [0.02 1.12]
Vermis VII	0.22 [-0.05 0.47]	0.06 [-0.55 0.38]
Vermis VIII	0.06 [-0.47 0.56]	0.05 [-0.42 0.52]
Vermis X	0.05 [-0.16 0.26]	0.03 [-0.25 0.27]

Abbreviations: CI = confidence interval

Supplementary Table 2. Sex-stratified means of standardized β_{age} across fusion regions.

Functional Fusion Regions	Male (Mean [95% CI])	Female (Mean [95% CI])
A1L	0.38 [0.25 0.49]	0.3 [0.21 0.38]
A1R	0.36 [0.24 0.49]	0.32 [0.12 0.32]
A2L	0.3 [0.18 0.43]	0.16 [0.06 0.25]
A2R	0.22 [0.1 0.34]	0.14 [0.04 0.24]
A3L	0.33 [0.22 0.45]	0.21 [0.12 0.29]
A3R	0.28 [0.17 0.4]	0.2 [0.11 0.31]
D1L	0.53 [0.41 0.64]	0.49 [0.26 0.46]
D1R	0.46 [0.34 0.58]	0.34 [0.14 0.35]
D2L	0.35 [0.23 0.46]	-0.12 [-0.20 0.33]
D2R	0.38 [0.26 0.48]	0.18 [0.08 0.28]
D3L	0.23 [0.11 0.36]	0.13 [0.02 0.23]
D3R	0.43 [0.32 0.55]	0.27 [0.17 0.37]
D4L	0.23 [0.11 0.36]	0.14 [0.04 0.23]
D4R	0.48 [0.38 0.59]	0.3 [0.2 0.4]
M1L	0.45 [0.34 0.55]	0.28 [0.18 0.37]
M1R	0.39 [0.28 0.52]	0.28 [0.17 0.38]
M2L	-0.21 [-0.32 0.45]	0.12 [0.02 0.22]
M2R	0.22 [0.1 0.35]	-0.11 [-0.20 0.22]
M3L	0.4 [0.28 0.51]	0.20 [0.08 0.28]
M3R	0.13 [0 0.24]	0.03 [-0.07 0.14]
M4L	0.03 [-0.05 0.41]	0.13 [0.04 0.22]
M4R	0.16 [0.04 0.28]	0.08 [-0.02 0.18]
S1L	0.53 [0.42 0.63]	0.31 [0.22 0.4]
S1R	0.4 [0.27 0.51]	0.18 [0.07 0.28]
S2L	0.41 [0.39 0.62]	0.54 [0.24 0.45]
S2R	0.29 [0.19 0.41]	0.09 [0 0.19]
S3L	0.46 [0.34 0.58]	-0.08 [-0.18 0.38]
S3R	0.28 [0.17 0.4]	0.11 [0.01 0.22]
S4L	0.32 [0.2 0.43]	0.17 [0.07 0.27]
S4R	0.3 [0.19 0.41]	-0.17 [-0.07 0.27]
S5L	0.11 [-0.01 0.22]	0.04 [-0.06 0.14]
S5R	0.17 [0.04 0.28]	0.1 [0.01 0.2]

Abbreviations: CI = confidence interval; A = Action; D = Demand; M = Motor; S = Socio-Linguistic; L = left; R = right.

Supplementary Table 3. Sex-stratified means of standardized β_{age} across MDTB regions.

MDTB Regions	Male (Mean [95% CI])	Female (Mean [95% CI])
1 (Left-Hand Presses)	0.31 [0.18 0.44]	0.11 [0.02 0.22]
2 (Right-Hand Presses)	0.16 [0.04 0.29]	0.08 [-0.02 0.18]
3 (Saccades)	0.31 [0.16 0.41]	0.26 [0.16 0.35]
4 (Action Observation)	0.31 [0.19 0.43]	0.23 [0.13 0.33]
5 (Divided Attention Left)	0.42 [0.29 0.54]	0.3 [0.19 0.4]
6 (Divided Attention Right)	0.19 [0.11 0.4]	0.12 [0.09 0.32]
7 (Narrative)	0.49 [0.38 0.59]	0.34 [0.25 0.43]
8 (Word Comprehension)	0.26 [0.15 0.38]	0.18 [0.08 0.27]
9 (Verbal Fluency)	0.27 [0.15 0.38]	0.16 [0.05 0.26]
10 (Autobiographical Recall)	0.39 [0.23 0.46]	0.3 [0.2 0.4]

Abbreviations: CI = confidence interval.

Supplementary Table 4: Sex-stratified means of standardized β_{age} across resting-state regions.

Resting-state regions	Male (Mean [95% CI])	Female (Mean [95% CI])
Somatomotor	0.15 [0.10 0.39]	0.21 [0.11 0.31]
Dorsal Attention	0.39 [0.27 0.5]	0.26 [0.16 0.36]
Ventral Attention	0.4 [0.28 0.51]	0.25 [0.16 0.35]
Limbic	0.12 [0.09 0.32]	0.22 [0.02 0.22]
Frontoparietal	0.23 [0.32 0.55]	0.35 [0.24 0.44]
Default	0.37 [0.25 0.47]	0.22 [0.15 0.35]

Abbreviations: CI = confidence interval.

Supplementary Table 5. Lobular region model comparison via leave-one-out cross-validation (LOOCV).

Lobular Regions	Linear Model LOOCV [SE]	B-Spline Model LOOCV [SE]	Difference [SE of difference]
Corpus Medullare	-665.82 [22.38]	-647.33 [21.04]	18.49 [4.64]
Left Crus I	-770.53 [22.63]	-766.98 [22.15]	3.54 [2.44]
Left Crus II	-794.2 [18.42]	-790.58 [18.2]	3.62 [2.98]
Left I-III	-115.1 [32.82]	-116 [32.55]	0.9 [1.89]
Left IV	-750.41 [19.69]	-747.2 [19.41]	3.21 [2.48]
Left IX	-809.4 [18.09]	-807.33 [18]	2.08 [2.22]
Left V	-727.11 [17.86]	-725.86 [17.93]	1.25 [2.96]
Left VI	-769.62 [21.49]	-769.88 [21.45]	0.26 [2.12]
Left VIIB	-721.83 [18.22]	-713.76 [18.53]	8.07 [4.36]
Left VIIIA	-795.63 [19.68]	-795.98 [19.61]	0.35 [1.87]
Left VIIIB	-762.07 [19.78]	-763.21 [19.87]	1.14 [1.41]
Left X	-828 [20.13]	-830.26 [20.27]	2.26 [1.35]
Right Crus I	-761.26 [19.76]	-758.19 [19.62]	3.06 [2.47]
Right Crus II	-788.1 [18.68]	-780.34 [18.41]	7.76 [3.79]
Right I-III	-810.84 [17.4]	-808.88 [17.62]	1.96 [2.4]
Right IX	-814.71 [16.79]	-813.08 [16.74]	1.63 [2.18]
Right V	-716.06 [28.06]	-709.08 [25.54]	6.98 [4.96]
Right VI	-748.76 [16.3]	-741.88 [16.22]	6.88 [3.48]
Right VIIB	-753.64 [17.05]	-748.1 [17.52]	5.54 [3.78]
Right VIIIA	-795.09 [19.56]	-792.47 [19.3]	2.62 [2.49]
Right VIIIB	-753.95 [18.67]	-753.88 [18.82]	0.07 [2.03]
Right X	-821.77 [20.95]	-821.48 [20.52]	0.28 [2.35]
Right IV	-781.38 [17.98]	-784.03 [18.04]	2.65 [1.05]
Vermis IX	-774.66 [26.45]	-770.02 [25.82]	4.64 [2.96]
Vermis VI	-728.81 [23.38]	-729.05 [23.24]	0.24 [2.34]
Vermis VII	-333.64 [33.03]	-333.24 [32.98]	0.4 [2.35]
Vermis VIII	-697.9 [24.19]	-696.18 [23.75]	1.72 [2.96]
Vermis X	-230.5 [33.09]	-228.2 [33.58]	2.29 [3.03]

Abbreviations: SE = standard error of the mean.

Supplementary Table 6. Functional fusion region model comparison via leave-one-out cross-validation (LOOCV).

Fusion Regions	Linear Model LOOCV [SE]	B-Spline Model LOOCV [SE]	Difference [SE of difference]
A1L	-646.63 [17.54]	-643.58 [17.6]	3.05 [3.35]
A1R	-685.45 [15.42]	-676.76 [15.74]	8.69 [4.97]
A2L	-663.33 [16.53]	-664.58 [16.64]	1.25 [2.02]
A2R	-693.69 [15.3]	-694.01 [15.71]	0.31 [1.67]
A3L	-645.26 [14.35]	-644.26 [14.52]	1 [2.7]
A3R	-701.75 [14.48]	-701.75 [14.48]	0 [1.87]
D1L	-649.23 [19.5]	-643.6 [20.27]	5.63 [4.24]
D1R	-674.56 [15.71]	-671.79 [15.65]	2.77 [3.42]
D2L	-660.23 [16.22]	-653.58 [16.4]	6.64 [4.16]
D2R	-660.6 [14.82]	-656.82 [15.17]	3.78 [3.87]
D3L	-693.3 [16.9]	-690.37 [17.24]	2.93 [3.3]
D3R	-667.2 [16.2]	-666.57 [16.51]	0.63 [2.63]
D4L	-682.59 [16.6]	-677.96 [16.67]	4.62 [3.36]
D4R	-660.46 [16.96]	-657.04 [17.44]	3.42 [3.21]
M1L	-639.76 [22.72]	-640.66 [22.62]	0.9 [2.05]
M1R	-680.67 [18.38]	-682.56 [18.29]	1.89 [1.73]
M2L	-681.22 [19.73]	-682.17 [19.5]	0.95 [1.91]
M2R	-701.76 [16.8]	-704.09 [16.85]	2.33 [1.32]
M3L	-678.22 [17.41]	-679.61 [17.6]	1.38 [2.19]
M3R	-715.15 [15.71]	-715.29 [15.58]	0.14 [2.3]
M4L	-650.37 [17.59]	-650.39 [17.53]	0.02 [2.41]
M4R	-695.99 [15.25]	-696.44 [15.17]	0.45 [1.7]
S1L	-611.45 [19.78]	-612.08 [19.58]	0.63 [2.49]
S1R	-691.66 [14.25]	-683.91 [14.11]	7.75 [4.88]
S2L	-650.93 [14.84]	-641.39 [14.96]	9.54 [4.9]
S2R	-685.85 [14.3]	-682.35 [15.16]	3.5 [3.75]
S3L	-653.88 [15.28]	-645.04 [15.1]	8.84 [5.07]
S3R	-689.04 [15.17]	-689.23 [15.27]	0.2 [2.29]
S4L	-678.55 [16.06]	-674.06 [16.34]	4.49 [3.59]
S4R	-653.07 [13.86]	-650.33 [14.36]	2.74 [3.53]
S5L	-694.11 [16.98]	-692.16 [16.5]	1.95 [3.14]
S5R	-701.69 [16.03]	-703.34 [15.97]	1.65 [1.15]

Abbreviations: SE = standard error of the mean; A = Action; D = Demand; M = Motor; S = Socio-Linguistic; L = left; R = right.

Supplementary Table 7. Functional MDTB region model comparison via leave-one-out cross-validation (LOOCV).

MDTB Regions	Linear Model LOOCV [SE]	B-Spline Model LOOCV [SE]	Difference [SE of difference]
1 (Left-Hand Presses)	-669.67 [15.65]	-672.24 [15.48]	2.57 [2.17]
2 (Right-Hand Presses)	-676.46 [14.21]	-678.76 [14.61]	2.31 [1.55]
3 (Saccades)	-662.75 [15.88]	-661.68 [15.85]	1.07 [2.59]
4 (Action Observation)	-646.35 [15.16]	-641.6 [15.85]	4.75 [4.4]
5 (Divided Attention Left)	-656.98 [15.71]	-651.02 [15.82]	5.96 [4.24]
6 (Divided Attention Right)	-661.2 [15.04]	-660.45 [15.64]	0.74 [2.74]
7 (Narrative)	-628.46 [18.35]	-627.62 [18]	0.84 [3.27]
8 (Word Comprehension)	-649.23 [13.42]	-645.02 [13.88]	4.2 [3.69]
9 (Verbal Fluency)	-666.14 [14.44]	-666.13 [14.82]	0.01 [2.5]
10 (Autobiographical Recall)	-641.19 [16.01]	-631.63 [16.15]	9.57 [4.74]

Abbreviations: SE = standard error of the mean.

Supplementary Table 8. Functional resting-state region model comparison via leave-one-out cross-validation (LOOCV).

Resting-State Regions	Linear Model LOOCV [SE]	B-Spline Model LOOCV [SE]	Difference [SE of difference]
Somatomotor	-667.48 [15.23]	-666.07 [15.06]	1.41 [2.69]
Dorsal Attention	-652.87 [14.61]	-639.9 [14.4]	12.97 [5.46]
Ventral Attention	-641.69 [14.1]	-634.59 [13.43]	7.1 [4.61]
Limbic	-674.66 [17.06]	-667.8 [16.71]	6.86 [4.53]
Frontoparietal	-648.15 [15.61]	-639.38 [15.72]	8.77 [4.66]
Default	-646.72 [14.06]	-633.08 [14.26]	13.64 [5.84]

Abbreviations: SE = standard error of the mean.

Supplementary Table 9. Significant PLS bootstrap ratios.

Variable Type	Variable	BSR	95% CI
Behavior	Language Comprehension	5.50	[0.04 0.09]
	Social Behavior	3.44	[0.02 0.07]
	Reading	3.18	[0.02 0.06]
Parcel	D2R	12.52	[0.17 0.24]
	D2L	10.38	[0.17 0.26]
	S4R	10.33	[0.17 0.25]
	S2R	10.02	[0.15 0.23]
	S3L	8.83	[0.16 0.25]
	S4L	8.74	[0.15 0.24]
	S2L	8.71	[0.15 0.24]
	D1R	8.15	[0.14 0.23]
	S3R	7.73	[0.14 0.23]
	D3L	7.43	[0.14 0.24]
	D3R	7.23	[0.12 0.21]
	A2L	7.04	[0.13 0.24]
	D4L	7.01	[0.14 0.24]
	M4L	6.99	[0.16 0.27]
	A1R	6.74	[0.12 0.23]
	S1R	6.59	[0.12 0.23]
	A2R	6.34	[0.11 0.21]
	D1L	6.15	[0.11 0.20]
	A1L	5.93	[0.12 0.23]
	M2L	5.81	[0.10 0.21]
	D4R	5.52	[0.10 0.22]
	M3R	5.22	[0.10 0.22]
	M3L	5.19	[0.09 0.19]
	M2R	4.90	[0.09 0.21]
	A3R	4.88	[0.10 0.24]
	S5R	4.71	[0.09 0.22]
	S5L	4.55	[0.09 0.24]
	S1L	4.45	[0.08 0.20]
	A3L	4.28	[0.09 0.23]
	M1L	4.03	[0.05 0.18]
	M4R	3.73	[0.08 0.23]

Variable Type	Variable	BSR	95% CI
	M1R	3.45	[0.05 0.19]

Only variables with |BSR| > 2 and 95% CI not crossing zero are shown. Variables are ordered by bootstrap ratio magnitude within each type. Abbreviations: CI = confidence interval; BSR = bootstrap ratio.

Supplementary Table 10. Wilcoxon signed-rank comparison of ElasticNet performance for cerebellum vs. other models (Desikan-Killiany cerebral atlas)

Behavior	Comparison	$M_{Cerebellum}$	$SD_{Cerebellum}$	M_{Other}	SD_{Other}	W	N	p	r
**Reading**	Cerebellum vs. Cerebral Cortex	-0.01	0.01	0.01	0.02	0.0	10	.006*	.81
	Cerebellum vs. Combined	-0.01	0.01	0.01	0.02	0.0	10	.002*	.87
**Language Comprehension**	Cerebellum vs. Cerebral Cortex	0.05	0.01	-0.01	0.01	0.0	10	.002*	.87
	Cerebellum vs. Combined	0.05	0.01	0.04	0.01	1.0	10	.002*	.84
**Social Behavior (SRS)**	Cerebellum vs. Cerebral Cortex	0.25	0.02	0.05	0.03	0.0	10	.002*	.87
	Cerebellum vs. Combined	0.25	0.02	0.18	0.04	0.0	10	.002*	.87

* = $p < .05$, two-sided
