# Supplementary material for: Cerebellar growth is associated with domain-specific cerebral maturation and socio-linguistic behavior
Source: Nat Commun. 2026 May 13;17:4338. doi: 10.1038/s41467-026-72940-5 (PMC13172492; doi:10.1038/s41467-026-72940-5)
Supplement: Supplementary file 2 — Reporting Summary [file 41467_2026_72940_MOESM2_ESM.pdf]

nature portfolio

Corresponding author(s): Aikaterina Manoli, MSc, and Sofie L. Valk, PhD

Last updated by author(s): March 5 2026

Reporting Summary

Nature Portfolio wishes to improve the reproducibility of the work that we publish. This form provides structure for consistency and transparency in reporting. For further information on Nature Portfolio policies, see our Editorial Policies and the Editorial Policy Checklist.

Statistics

For all statistical analyses, confirm that the following items are present in the figure legend, table legend, main text, or Methods section.

n/a	Confirmed	
☐	☒	The exact sample size (*n*) for each experimental group/condition, given as a discrete number and unit of measurement
☐	☒	A statement on whether measurements were taken from distinct samples or whether the same sample was measured repeatedly
☐	☒	The statistical test(s) used AND whether they are one- or two-sided<br>*Only common tests should be described solely by name; describe more complex techniques in the Methods section.*
☐	☒	A description of all covariates tested
☐	☒	A description of any assumptions or corrections, such as tests of normality and adjustment for multiple comparisons
☐	☒	A full description of the statistical parameters including central tendency (e.g. means) or other basic estimates (e.g. regression coefficient) AND variation (e.g. standard deviation) or associated estimates of uncertainty (e.g. confidence intervals)
☐	☒	For null hypothesis testing, the test statistic (e.g. *F*, *t*, *r*) with confidence intervals, effect sizes, degrees of freedom and *P* value noted<br>*Give P values as exact values whenever suitable.*
☐	☒	For Bayesian analysis, information on the choice of priors and Markov chain Monte Carlo settings
☐	☒	For hierarchical and complex designs, identification of the appropriate level for tests and full reporting of outcomes
☐	☒	Estimates of effect sizes (e.g. Cohen's *d*, Pearson's *r*), indicating how they were calculated

Our web collection on statistics for biologists contains articles on many of the points above.

Software and code

Policy information about availability of computer code

Data collection	The present study used existing developmental data from the Lifespan BCP (nda.nih.gov/edit_collection.html?id=2848) and the Lifespan 2.0 HCP-D release (nda.nih.gov/general-query.html?q=query=featured-datasets:HCP%20Aging%20and%20Development). Neuroimaging and behavioral data are publicly available for download from the provided links.
Data analysis	Image preprocessing leveraged open-source software (iBEAT V2.0: github.com/iBEAT-V2/iBEAT-V2.0-Docker; HCP minimal preprocessing pipeline: github.com/Washington-University/HCPpipelines). Parcellations for the cerebellum and the cerebral cortex were also derived by openly available tools (ACAPULCO: gitlab.com/shuohan/acapulco; FSL: fsl.fmrib.ox.ac.uk/fsl/docs/#/; FreeSurfer: surfer.nmr.mgh.harvard.edu). ITK-SNAP for manual correction of segmentations is freely available online (itksnap.org/pmwiki/pmwiki.php). Normative modeling code is available via the PCNtoolkit (github.com/amarquand/PCNtoolkit). Scripts for all processing and analysis pipelines used in this study can be found on GitHub (github.com/kmanoli/NormCerebellum).

For manuscripts utilizing custom algorithms or software that are central to the research but not yet described in published literature, software must be made available to editors and reviewers. We strongly encourage code deposition in a community repository (e.g. GitHub). See the Nature Portfolio guidelines for submitting code & software for further information.

Data

Policy information about availability of data

All manuscripts must include a data availability statement. This statement should provide the following information, where applicable:

- Accession codes, unique identifiers, or web links for publicly available datasets
- A description of any restrictions on data availability
- For clinical datasets or third party data, please ensure that the statement adheres to our policy

The present study used existing developmental data from the Lifespan BCP (nda.nih.gov/edit_collection.html?id=2848) and the Lifespan 2.0 HCP-D release (nda.nih.gov/general-query.html?q=query=featured-datasets:HCP%20Aging%20and%20Development). Neuroimaging and behavioral data are publicly available for download from the provided links. The cerebellar functional atlases are available on GitHub (github.com/DiedrichsenLab/cerebellar_atlases). The cerebellar growth models for lobular and functional parcels are also available on GitHub (github.com/kmanoli/NormCerebellum).

Research involving human participants, their data, or biological material

Policy information about studies with human participants or human data. See also policy information about sex, gender (identity/presentation), and sexual orientation and race, ethnicity and racism.

Reporting on sex and gender	Findings apply to both sexes (biologically defined via self-report). We provide sex-specific growth trajectories of cerebellar and cerebral parcels. The study included 354 biological male and 397 biological female participants. Gender (socially and culturally defined) was not considered.
Reporting on race, ethnicity, or other socially relevant groupings	Our analyses did not control for any social groupings. Our analyses only controlled for age and biological sex.
Population characteristics	We analyzed data of 751 typically developing infants, children, and adolescents (age: 1-21 years; M = 12.86, SD = 5.49; 397 female). Participants had no history of psychiatric or neurodevelopmental disorders or other relevant conditions.
Recruitment	The present study used existing developmental data from the Lifespan BCP (nda.nih.gov/edit_collection.html?id=2848) and the Lifespan 2.0 HCP-D release (nda.nih.gov/general-query.html?q=query=featured-datasets:HCP%20Aging%20and%20Development).
Ethics oversight	This is a secondary analysis of existing, openly available data. Ethical approval for data collection was granted at the relevant institutions. MPI for Human Cognitive and Brain Sciences does not require additional approval for the analysis of openly available data.

Note that full information on the approval of the study protocol must also be provided in the manuscript.

Field-specific reporting

Please select the one below that is the best fit for your research. If you are not sure, read the appropriate sections before making your selection.

[x] Life sciences   [ ] Behavioural & social sciences   [ ] Ecological, evolutionary & environmental sciences

For a reference copy of the document with all sections, see nature.com/documents/nr-reporting-summary-flat.pdf

Life sciences study design

All studies must disclose on these points even when the disclosure is negative.

Sample size	We conducted our analyses starting from the first year of life as the cutoff point. In these scans, cerebellar lobular folds were visible and image processing and segmentation algorithms performed reasonably well. After exclusions due to poor image quality, we analyzed a final sample of 751 participants (age: M = 12.86, SD = 5.49; 397 female). Thus, our sample size was not predetermined, but depended on the quality of available data. Note that cerebellar segmentations were manually corrected, which was a laborious process that nonetheless resulted in high-quality estimates of cerebellar volume.
Data exclusions	Overall, we excluded 94 BCP and 12 HCP-D participants due to poor image quality, insufficient cerebellar coverage, or poor performance of the segmentation algorithms after visual inspection among three independent reviewers. We further excluded 11 participants due to artifacts in cerebral cortical surface reconstruction.
Replication	For normative modeling, participants were randomly split into train (80%) and test (20%) sets, keeping the batch effects proportional. All analyses further included cross-validation to estimate how our models perform on unseen data. Where appropriate, we further applied bootstrap resampling to assess the stability of the estimates and ensure that the results were not unduly influenced by sampling variability.
Randomization	For normative modeling, age was modeled as a covariate and sex and scanner site ad batch effects. Participants were randomly split into train (80%) and test (20%) sets, keeping the batch effects proportional. For behavioral associations, behaviors were residualized for age and sex.
Blinding	Blinding was not relevant because participants were not assigned to experimental groups. This is a cross-sectional investigation of normative cerebellar growth and how it relates to cerebral maturation and behavioral outcomes.

Reporting for specific materials, systems and methods

We require information from authors about some types of materials, experimental systems and methods used in many studies. Here, indicate whether each material, system or method listed is relevant to your study. If you are not sure if a list item applies to your research, read the appropriate section before selecting a response.

Materials & experimental systems

n/a	Involved in the study	
☒	☐	Antibodies
☒	☐	Eukaryotic cell lines
☒	☐	Palaeontology and archaeology
☒	☐	Animals and other organisms
☒	☐	Clinical data
☒	☐	Dual use research of concern
☒	☐	Plants

Methods

n/a	Involved in the study	
☒	☐	ChIP-seq
☒	☐	Flow cytometry
☐	☒	MRI-based neuroimaging

Plants

Seed stocks

Report on the source of all seed stocks or other plant material used. If applicable, state the seed stock centre and catalogue number. If plant specimens were collected from the field, describe the collection location, date and sampling procedures.

Novel plant genotypes

Describe the methods by which all novel plant genotypes were produced. This includes those generated by transgenic approaches, gene editing, chemical/radiation-based mutagenesis and hybridization. For transgenic lines, describe the transformation method, the number of independent lines analyzed and the generation upon which experiments were performed. For gene-edited lines, describe the editor used, the endogenous sequence targeted for editing, the targeting guide RNA sequence (if applicable) and how the editor was applied.

Authentication

Describe any authentication procedures for each seed stock used or novel genotype generated. Describe any experiments used to assess the effect of a mutation and, where applicable, how potential secondary effects (e.g. second site T-DNA insertions, mosiacism, off-target gene editing) were examined.

Magnetic resonance imaging

Experimental design

Design type

N/A

Design specifications

N/A

Behavioral performance measures

Out-of-scanner behavioral tasks in the National Institutes of Health (NIH) Toolbox (nihtoolbox.org).

Acquisition

Imaging type(s)

Structural (T1w) images

Field strength

3 Tesla

Sequence & imaging parameters

All data were acquired with 3T Siemens Prisma MRI scanners (Siemens, Erlangen, Germany) with 32 channel head coils. Younger participants in the BCP were scanned while naturally asleep without the use of sedatives. T1-weighted (T1w) images were acquired with a 3D MPRAGE sequence with the following parameters: sagittal field of view of 256×240×166mm with a matrix size of 320×300×208 slices, resolution of 0.8mm isotropic voxels, and flip angle of 8 degrees. The TR/TE parameters for BCP and HCP-D T1w images are 2400/2.24 ms and 2500/2.22 ms, respectively.

Area of acquisition

Whole-brain

Diffusion MRI ☐ Used ☒ Not used

Preprocessing

Preprocessing software

HCP-D images were already preprocessed according to the HCP minimal preprocessing pipeline. Specifically, we utilized native space T1w images that had been aligned to T2w images and undergone bias field correction. Quality-checked BCP images were minimally preprocessed using iBEAT V2.0, a state-of-the-art pipeline specifically optimized for infant and toddler MRI data. T1w and T2w images were reoriented to a consistent left-posterior-inferior (LPI) orientation, underwent bias field correction, and were subsequently aligned to each other.

Normalization

T1w images were analyzed in subjects' space to capture subject-specific anatomical variability, which is essential for constructing accurate normative models of brain volume.

Normalization template | N/A

Noise and artifact removal | Participants were excluded for incomplete cerebellar coverage and poor image quality (after visual inspection). Participants were further excluded for performance of cerebellar segmentation algorithms (after visual inspection and if data were over 2DSs of the mean) and cerebral segmentation algorithms (Euler index (EI) > |2| median deviations from the dataset-specific median EI).

Volume censoring | N/A

Statistical modeling & inference

Model type and settings

Normative modeling: We generated normative models for lobular and functional cerebellar and cerebral cortical subregions using Hierarchical Bayesian Regression (HBR), with age as a covariate and sex and site as batch effects. We accommodated skewed distributions through the sinh-arcsinh likelihood (SHASHb) and modeled random effects in intercept, slope, and variance (sigma) on the batch-effects (sex and site). We evaluated out-of-sample predictive performance of both model types using leave-one-out cross-validation.

Associations with cerebral cortex: We employed regularized regression to examine associations between the normative model z-scores of cerebellar and cerebral parcels, predicting cerebral z-scores from cerebellar z-scores. Model selection and evaluation were performed using nested cross-validation to prevent overfitting during hyperparameter selection, and Ridge model demonstrated the highest performance, measured via R2. Statistical significance of individual cerebellar-cerebral associations was assessed via 10,000 permutations.

Associations with behavioral outcomes: We used partial least squares (PLS) analysis to associate participants' normative model-derived z-scores for each cerebellar parcel with their behavioral scores in the HCP-D dataset. We evaluated up to ten latent variables using permutation testing (10,000 iterations). To assess stability of the PLS approach, we applied bootstrap resampling (10,000 iterations) and calculated 95% CIs around the loading weights of parcel z-scores and behaviors. Lastly, we employed 10-fold cross-validation on the PLS model. As a final step, we assessed how cerebellar normative growth was associated with each behavior in relation to cerebral growth. Cerebellar and cerebral parcels were analyzed separately and in combination by concatenating their respective features. For each behavioral outcome, we employed regularized regression to associate individual participants' parcel z-scores with their behavioral scores. Model selection and evaluation was performed in the same way as in the cerebral associations, with ElasticNet performing better on average across behaviors and feature sets. Performance differences between cerebellar-only, cerebral cortex-only, and combined feature sets were evaluated using pairwise Wilcoxon signed-rank tests across the ten cross-validation folds.

Effect(s) tested

Normative model outputs include posterior distributions of the parameters and individual deviations from the normative range (z-scores). Z-scores for cerebellar and cerebral parcels were then associated via Ridge regression to identify co-maturation patterns. Lastly, cerebellar z-scores were associated with behavioral task performance.

Specify type of analysis: ☐ Whole brain ☒ ROI-based ☐ Both

Anatomical location(s)

Lobular ROIs: ACAPULCO (Han et al., 2020)
Functional ROIs: MDTB (King et al., 2010), functional fusion (Nettekoven et al., 2024), resting-state (Buckner et al., 2011).

Statistic type for inference (See Eklund et al. 2016) | Structural ROI-level inference.

Correction | Cerebello-cerebral associations were corrected with FDR (q = .05).

Models & analysis

n/a	Involved in the study
☒	☐ Functional and/or effective connectivity
☒	☐ Graph analysis
☐	☒ Multivariate modeling or predictive analysis

Multivariate modeling and predictive analysis

Normative modeling: HBR model. Independent variables: age, sex, scanner site. Dependent variables: Cerebellar parcel grey matter volumes. Data split: 80% training, 20% test, keeping sex and site proportional in each set. Evaluation: Leave-one-out cross-validation. Parcels were modeled separately.

Associations with cerebral cortex: Model: Ridge regularized regression. Independent variable: Cerebellar parcel normative z-scores. Dependent variable: Cerebral parcel normative z-scores. Evaluation: 10-fold cross-validation.

Associations with behavioral outcomes: 1) Model: PLS. Variables: Cerebellar parcel normative z-scores and behavioral outcomes. Evaluation: 10-fold cross-validation and bootstrap resampling (10,000 iterations). 2) Model: ElasticNet regularized regression. Independent variables: Normative z-scores of cerebellar, cerebral, and combined cererbello-cerebral parcels (feature sets were modeled separately). Dependent variables: Socio-linguistic behavioral outcomes (modeled separately). Evaluation: 10-fold cross-validation.
